# Discovery of Metabolic Biomarkers for Duchenne Muscular Dystrophy within a Natural History Study

**DOI:** 10.1371/journal.pone.0153461

**Published:** 2016-04-15

**Authors:** Simina M. Boca, Maki Nishida, Michael Harris, Shruti Rao, Amrita K. Cheema, Kirandeep Gill, Haeri Seol, Lauren P. Morgenroth, Erik Henricson, Craig McDonald, Jean K. Mah, Paula R. Clemens, Eric P. Hoffman, Yetrib Hathout, Subha Madhavan

**Affiliations:** 1 Innovation Center for Biomedical Informatics, Georgetown University Medical Center, Washington, DC, United States of America; 2 Department of Oncology, Georgetown University Medical Center, Washington, DC, United States of America; 3 Department of Biostatistics, Bioinformatics, and Biomathematics, Georgetown University Medical Center, Washington, DC, United States of America; 4 Department of Biochemistry and Molecular & Cellular Biology, Georgetown University Medical Center, Washington, DC, United States of America; 5 Children’s National Medical Center and the George Washington University, Washington, DC, United States of America; 6 Department of Physical Medicine and Rehabilitation, University of California Davis, School of Medicine, Davis, California, United States of America; 7 Department of Pediatrics, University of Calgary, Alberta Children’s Hospital, Calgary, Alberta, Canada; 8 Neurology Service, Department of Veteran Affairs Medical Center, Pittsburgh, Pennsylvania, United States of America; 9 Department of Neurology, University of Pittsburgh, Pittsburgh, Pennsylvania, United States of America; Rutgers University -New Jersey Medical School, UNITED STATES

## Abstract

Serum metabolite profiling in Duchenne muscular dystrophy (DMD) may enable discovery of valuable molecular markers for disease progression and treatment response. Serum samples from 51 DMD patients from a natural history study and 22 age-matched healthy volunteers were profiled using liquid chromatography coupled to mass spectrometry (LC-MS) for discovery of novel circulating serum metabolites associated with DMD. Fourteen metabolites were found significantly altered (1% false discovery rate) in their levels between DMD patients and healthy controls while adjusting for age and study site and allowing for an interaction between disease status and age. Increased metabolites included arginine, creatine and unknown compounds at m/z of 357 and 312 while decreased metabolites included creatinine, androgen derivatives and other unknown yet to be identified compounds. Furthermore, the creatine to creatinine ratio is significantly associated with disease progression in DMD patients. This ratio sharply increased with age in DMD patients while it decreased with age in healthy controls. Overall, this study yielded promising metabolic signatures that could prove useful to monitor DMD disease progression and response to therapies in the future.

## Introduction

Duchenne muscular dystrophy is an X-linked genetic disease and the most common childhood neuromuscular disorder, with an incidence of about 1 in 5,000 newborn males [1–3]. The disease is characterized by a natural history consisting of progressive muscle degeneration, loss of ambulation by the age of 12, and eventually death by late 20s to early 30s [4, 5]. DMD is caused by mutations in the dystrophin gene resulting in complete loss of the dystrophin protein that maintains muscle fiber integrity and function [6]. Absence of dystrophin causes muscle fragility and loss of muscle fibers which are replaced by fat and connective tissue. While understanding of the disease and clinical management–including the use of glucocorticoid treatment and assisted ventilation–has continued to improve over the last few decades [7, 8], development of novel therapies and testing of novel drugs is still hindered by the small numbers of patients available for clinical trials and the difficulties in selecting appropriate outcome measures [9]. As a result, regulatory agencies such as the Food and Drug Administration (FDA) and European Medicines Agency (EMA) are more willing to consider surrogate biomarkers as endpoint measures in Phase II dose-ranging studies. Thus it is important to find and identify reliable biomarkers associated with disease progression and response to therapies for DMD patients.

The best-known serum molecular biomarker for DMD is muscle-derived creatine kinase (CK) [10], which is typically measured by enzymatic activity. Serum elevations of CK are generally considered a diagnostic biomarker of DMD, even in presymptomatic infants [11]. However, the large inter- and intra-person variability in CK levels, partly caused by the impact of the child’s age and sensitivity to physical activity, makes it a poor candidate for use as a surrogate biomarker. Furthermore, CK levels have been shown to be inversely correlated with disease progression and severity due to the loss of muscle tissue for more advanced patients, which causes less CK to leak into the blood stream and be detected [12].

In the last few years, there has been renewed interest in defining biochemical biomarkers for DMD. These include miRNAs [13–15] and proteins [12, 16–20], but little research has been dedicated to identifying metabolic biomarkers for DMD [21].

Metabolites are small molecular mass components or intermediate products of metabolism that can be detected in biofluids and tissues; they regulate and maintain physiology homeostasis and have various biological functions. They can be influenced by genetics, as well as by environmental factors [22]. Metabolites are easily measurable using high throughput technologies and can be transitioned into clinical assays. They are widely used in other diseases (e.g. serum creatinine is a marker of liver function) but had very limited implementation in DMD. “Metabolomics” refers to the systematic assessment of metabolites. Metabolic profiles have been used to predict disease risk, to diagnose disease, or as biomarkers of disease in a variety of disorders, including diabetes, prostate cancer, and Crohn’s disease [23–26]. Untargeted metabolomics approaches in DMD can add to the growing catalogue of protein and miRNA biomarkers which may be used to monitor disease progression and, as a result, to evaluate therapeutic response. The present study proposes several such putative metabolic biomarkers.

## Materials and Methods

### Study participants and samples

We analysed the circulating serum metabolites of 51 DMD patients and 22 healthy volunteers from 5 different study sites enrolled through the Cooperative International Neuromuscular Research Group (CINRG) Duchenne Natural History Study. All the study participants were male. The cases and controls were age-matched, with some enrichment for older cases. Summary characteristics of the study participants are provided in Table 1 and detailed in S1 Table. The DMD patients had a minimum age of 4, a maximum age of 28.7, and a median age of 11.4 years. The healthy controls had a minimum age of 6, a maximum age of 17.8, and a median age of 13.7 years. The study protocol was approved by Institutional Review Boards at all participating institutions, and informed written consent was obtained from participants or their parent or legal guardian. The Institutional Review Boards were: Conjoint Health Research Ethics Board (CHREB) (for Alberta Children’s Hospital, Calgary), UC Davis Institutional Review Board, University of Pittsburgh Institutional Review Board, Children’s National Medical Center Institutional Review Board (CNMC IRB) and Executive Committee of the Sydney Children’s Hospitals Network (SCHN) Human Research Ethics Committee (HREC) (for Children’s Hospital at Westmead in Australia).

**Table 1:** Summary characteristics of study participants.

Study site	DMD patients	Healthy controls
Alberta Children’s Hospital (Calgary)	19	16
University of California, Davis (UC Davis)	26	0
University of Pittsburgh / Children's Hospital of Pittsburgh of UPMC (U of Pittsburgh)	5	2
Children’s National Medical Center (CNMC)	0	4
Children’s Hospital at Westmead (Australia)	1	0
**Median age in years (minimum, maximum)**	11.4 (4, 28.7)	13.7 (6, 17.8)
**Total by age category**		
4–7 years	15	2
> 7–11 years	8	3
> 11–18 years	17	17
> 18–29 years	11	0
**Total**	51	22

For both DMD patients and healthy controls, 15 mL of blood was taken according to the study protocol. The blood was separated into 3 red top tubes, which were then mixed by 5 complete inversions. Samples were allowed to clot for 30 minutes at room temperature in a vertical position. After clotting, the tubes were centrifuged for 10 minutes to separate the serum, which was carefully collected by avoiding contact with the blood clot. The serum was then aliquoted into four polypropylene Cryogenic vials from Thermo Scientific Nalgene. Each transfer tube of 500 μL was frozen in a secure -80°C freezer until shipment. Samples were not thawed and refrozen until analysis.

### Liquid chromatography-mass spectrometry (LC-MS) analysis

For metabolite extraction, a single technical replicate for each serum sample was processed by adding 175 μl extraction buffer (A solution of 40% Acetonitrile, 25% Methanol and 35% Water containing internal standards [10μl of 1mg/ml debrisoquine and 50μl of 1mg/ml 4- nitrobenzoic acid]) to 25 μl of each serum sample. The samples were incubated on ice for 10 minutes and centrifuged at 13,000 rpm for 20 minutes at 4°C. The supernatant was transferred to a fresh Eppendorf vial and dried under vacuum. The dried samples were reconstituted in 200 μl of 5% Methanol, 1% Acetonitrile and 94% water solution. The samples were re-centrifuged at 13,000 rpm for 20 minutes at 4°C and supernatant transferred to fresh vials for UPLC- QTof analysis. 2μl of each sample was injected onto a Waters Acquity CSH C18 1.7 μm, 2.1 × 100mm column using an Acquity UPLC system by Waters Corporation, Milford, MA. The gradient mobile phase consisted of Solvent A- 100% water with 0.1% formic acid, Solvent B- 100% acetonitrile with 0.1% Formic acid and Solvent D- 9:1 ratio of Isopropanol to Acetonitrile with 0.1% Formic acid and 10mM Ammonium Formate. To reduce the chance of possible batch effects, the cases and controls were randomized. Each sample was run onto the column for 13 minutes at a flow rate of 0.4 ml per minute. The column temperature was set to 60° C. The gradient consisted of 97% Solvent A for 3 minutes and then at a ramp of curve 6 to 60% Solvent B from 0.5 to 4 minutes. From 4.0 to 8.0 minutes at a ramp of curve 6, the gradient moved to 98% of solvent B and at 9 minutes shifting to 5% Solvent B and 95% solvent D for10 minutes. At 11 minutes, the gradient was 25% Solvent A, 25% solvent B and 50% solvent D at a curve of ramp 6 and then back to initial conditions of 97% solvent A and 3% solvent B at 13 minutes. The elution from the column was introduced to Quadrupole Time of flight Mass spectrometer (Waters G2- Qtof) by electrospray Ionization in both positive and negative mode at a capillary voltage of 3.5 kV and sampling cone voltage of 35 V. The source temperature was set to 120° C and Desolvation temperature to 350 °C. The cone gas flow was maintained at 25L/hr and Desolvation gas flow at 750L/hr. Leucine- Encephalin solution in 50% acetonitrile was used a reference mass ([M+H]^+^ = 556.2771 and [M-H]^−^ = 554.2615) to maintain accurate mass. The data was acquired in centroid mode from 50 to 1200 mass range with the software Mass lynx (Waters Corporation). The column was initially conditioned by multiple injections of pooled quality controls and then every ten injections subsequently. The mass accuracy was monitored by injecting a mixture of standard compounds at the beginning and at the end of the batch.

### Data processing

The resulting CDF files were processed together using the XCMS method [27] to align the peaks and estimate the metabolite intensities for each sample. The exact steps performed were: feature detection, retention time correction, alignment of peaks into peak groups, and imputation of the values for the missing peaks based on the raw files. Thus, each peak group may have slightly different m/z values in different samples. The groups which had fewer than 19 peaks detected across all samples prior to imputation were removed. Henceforth, we will refer to “peak groups” as simply “peaks.” Prediction of isotopes was then performed using the CAMERA package [28] and higher-weight isotopes were removed. This resulted in 313 peaks in the negative mode and 1892 peaks in the positive mode, including nitrobenzoic acid and debrisoquine, respectively. Four of the estimated peak intensities, corresponding to two peaks, were equal to zero; we imputed these as the minimum non-zero value for that peak across all samples. The intensities were then normalized to the intensities of the two internal standards, quantile normalized (separately for the positive and negative modes), then log transformed (we used a log_2_ basis, but the results do not depend on the basis). Thus, a total of 2203 peaks were considered in downstream analyses. Individual-level internal standard normalized, quantile normalized, and log2-transformed peak intensity values are given in S1 Table.

### Statistical and bioinformatic analysis

#### Variables considered

The primary variable of interest for all the analyses considered below was DMD disease status. Additional variables considered were: age, age-by-DMD status multiplicative interaction, and study site. We included the effect of age, as metabolites related to hormonal and other physical changes in individuals between childhood and adulthood are expected to be reflected in metabolomics measurements, even in healthy controls. Other sources of variation not related to the disease process might exist. For example, many metabolites show associations with diet [29], some of which might also change with age. Since DMD is a progressive disorder, the age trends may be different in cases and controls, hence the use of the age-by-DMD status interaction term. We considered the effect of the study site, since demographic characteristics and environment factors including altitude or latitude might be different in the populations served by the different centers. Furthermore, we wished to account for any effects due to technical variables which were not controlled for between sites and which might have an impact on the results. For example, issues like personnel differences could play an important role and can never be fully eliminated [30].

#### Global exploratory analyses

A principal components analysis (PCA) was also performed as an exploratory analysis to check for possible technical artifacts and any other unusual patterns. After the PCA, 9 linear regression models were considered for the first principal component (PC1) to perform an assessment of variables which may have a “global” impact on the metabolite profiles. These models included as explanatory variables all possible combinations of DMD status, age, and site, as well as the possible age-by-DMD status interaction, only considered in models where both age and DMD status were also included separately. The best-fitting model in terms of the trade-off between model fit and number of parameters were chosen by the Akaike Information Criterion (AIC).

#### Linear regression analyses

The goal of the main statistical analysis was to find metabolites that are significantly different between DMD patients and healthy controls. The following linear model was fit to the transformed and normalized intensities:

$$Met=\beta_{0}+\beta_{1}x1(Status=DMD)+\beta_{2}xAge+\beta_{3}x1(Status=DMD)\;xAge+\beta_{site}+Noise,$$

where β_site_ is an intercept for each site with the exception of Australia, which serves as a baseline.

Equivalently, this means that for DMD patients, the model was:

$$Met=(\beta_{0}+\beta_{1})+(\beta_{2}+\beta_{3})xAge+\beta_{site}+Noise,$$

while for healthy controls, it was:

$$Met=\beta_{0}+\beta_{2}xAge+\beta_{site}+Noise.$$

As a result, testing the null of no effect of the DMD disease on the metabolite levels is equivalent, in this framework, to testing whether both the intercept and the slope for age are the same in the two groups:

$${H_{0}:\beta }_{1}=\beta_{3}=0.$$

We perform this F-test for each of the 2203 metabolite peaks, adjusting for multiple testing via the Bonferroni threshold, to ensure control of the familywise error rate (FWER) at a significance level of 0.05 and via the Benjamini-Hochberg (BH) correction [31]–which estimates conservative q-values [32]–to ensure control of the false discovery rate (FDR) at a significance level of 0.01. The significant peaks from the FDR-adjusted analysis were annotated via the HMDB [22] and Metlin [33] databases and manually inspected. These peaks were also considered in receiver operator characteristic (ROC) curve analyses, which fit a logistic regression model with case/control status as outcome and age and peak intensity as explanatory variables.

Two further analyses were considered. One subgroup analysis considered the same linear model, but only on the 62 study participants with ages 4–18 years. This is because the DMD group contained adult patients, whereas the control group did not. A subgroup analysis was also performed for the study participants from Calgary, using the model:

$$Met=\beta_{0}+\beta_{1}x1(Status=DMD)+\beta_{2}xAge+\beta_{3}x1(Status=DMD)\;xAge+Noise$$

and performing the analogous F-test. The purpose of this analysis was to see whether the most significant peaks are also found in a subset which is expected to be more homogeneous, in terms of both demographics and sample processing. Note that the Calgary subgroup had the largest sample size and was also somewhat balanced in terms of cases and controls (Table 1).

#### Validation of significant metabolites

For validation studies, eight serum samples were processed and run for tandem mass spectrometry (MS/MS) on Waters Quadruple time of flight Mass Spectrometer with ultra-performance liquid chromatography (Waters G2- Qtof). Samples were processed in the similar manner as in the profiling experiment and the chromatographic conditions used for data acquisition remained the same. The MS/MS spectra obtained for the significant m/z was then used to match with available spectra in online databases for identification of the biomarkers.

#### Pathway analyses

We used the web-based resource ConsensusPathDB [34] to perform pathway over-representation analysis. ConsensusPathDB contains a total of 4349 pathways from 12 different pathway databases such as Reactome, Kegg, Signalink, Biocarta, Pharmgkb, Netpath, Smpdb, Inoh, Wikipathways, Pid, Ehmn and Humancyc. HMDB IDs of the peaks validated by MS/MS were used to conduct pathway enrichment analysis. For each pathway, the hypergeometric distribution was used to test for over-representation of the metabolites in the input list of validated peaks among the metabolites in the pathway set. A multiple testing correction was performed using the Benjamini-Hochberg approach to control the FDR for the pathways that overlapped with at least one of the input HMDB IDs.

#### Bayesian network analyses

A Bayesian network analysis was conducted to develop a classifier for the participants in the study using a minimal set of metabolites, for both the entire dataset and the Calgary subset. The goal of this analysis was to identify the minimum set of peaks that classify samples as either DMD or control. The full data analysis considered age, normalized peak intensities, and site. Age and the peak intensity values were discretized using density approximation with 3 bins. The Markov Blanket network learning algorithm [35] was then applied to create the network. The Calgary-only analysis considered age and normalized peak intensities.

We then performed five-fold cross validation using the Markov Blanket algorithm. The data was split randomly into 5 approximately equal subsets, with the learning being performed on 80% of the data and the remaining 20% being used for validation for each of the 5 combinations of learning/validation sets.

## Results

### Global exploratory analysis

**Fig 1:**
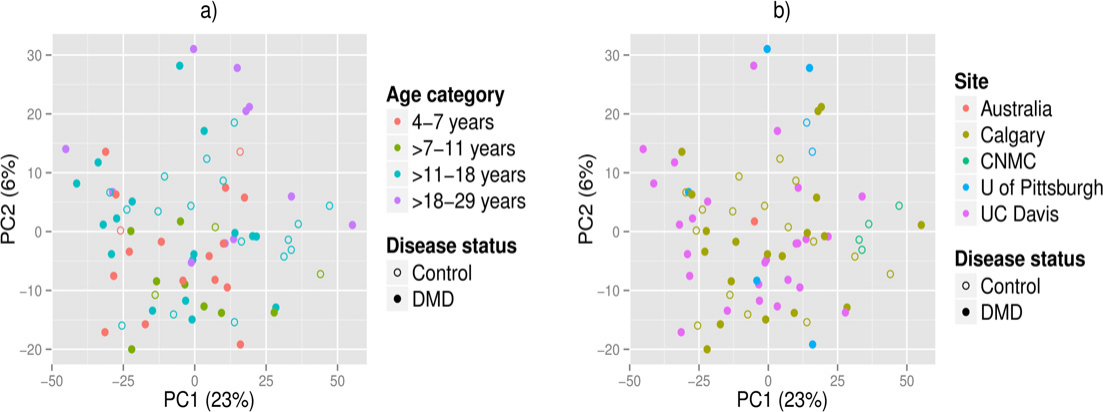
PCA plots of the internal standard normalized, quantile normalized, and log2-transformed intensity data. PC2 is plotted against PC1. Solid circles are DMD patients, empty circles are healthy controls. In panel a), individuals are color-coded by age category and in panel b) by study site.

The top two principal components are shown in Fig 1(A) and 1(B). The samples in Fig 1 are shape-coded by DMD disease status; in Fig 1(A) they are also color-coded by age category, whereas in Fig 1(B) they are color-coded by study site. 23% of the total variance is explained by the first principal component (PC1) and 6% by the second principal component (PC2). No clear clusters are observed. The regression models considered with PC1 as the outcome, along with the corresponding AIC and relative probability that they minimize the information loss [36] are presented in S2 Table. The top model selected via AIC for PC1 had age and study site, with study site being significant (p = 0.008 from F-test comparing it to model with only age) and age showing a borderline significant association (p = 0.066). Thus, both age and study site appear to have an impact on global metabolite profiles.

### Serum metabolome signature for DMD

Based on the F-tests performed for each of the 2203 peaks, testing for an effect of disease status in the presence of age and study site and allowing for an interaction between disease status and age, we find 14 metabolites to be statistically significant using the Benjamini-Hochberg procedure with q-value (adjusted p-value) ≤ 0.01, thus controlling the FDR at the 0.01 level. This means that we would expect the average fraction of false discoveries to be no more than 1% when repeatedly using this procedure. Of these 14 metabolites, 8 also showed statistically significant associations at a Bonferroni threshold of 0.05/2203, therefore controlling the FWER at 0.05. This means that using a p-value cut-off of 0.05/2203 for rejection for each peak, we would expect to obtain at least one false discovery (a peak which is not truly different between cases and controls but for which the null is rejected) no more than 5% of the time when repeatedly using this procedure on individuals sampled in the same way. Significant differences indicate that the fitted age trends for the DMD and control groups differed in slope and/or intercept, i.e. either there is a difference between intensity levels in normal and DMD individuals at baseline, which is maintained at different ages (difference in intercept); or there is no difference at baseline, but the values change according to age (difference in slope); or there is a difference at baseline and the values change according to age as well (difference in intercept and slope.) We focused the metabolite annotation and validation efforts on these 14 peaks. Each peak was annotated using the HMDB and Metlin databases (see S3 Table for the positive mode and S4 Table for the negative mode). Following MS/MS validation (S1 Fig), the identities of 4 of these peaks were determined very likely to be: 5a-DHT; creatinine; either epitestosterone sulfate, dehydroepiandrosterone sulfate, or testosterone sulfate; and creatine, corresponding to adducts with median m/z values of 369.17, 114.07, 367.16, and 132.08 in the LC-MS experiment. The MS/MS spectra for these peaks were all in accordance with the most likely database annotations. The identity of a fifth peak was determined to likely be L-arginine upon manual inspection; the adduct for this peak has an m/z value of 174.15, which was close to the monoisotopic mass of 174.11 for L-arginine, however it was not identified in the database annotation.

A subgroup analysis was conducted for the participants aged 4–18 years. This retained 6 significant peaks (m/z values of 357.25, 449.15, 369.17, 114.07, 132.08, 312.01) at the Bonferroni threshold of 0.05/2203, with no additional peaks significant at the q-value threshold of 0.01. Five of these peaks (all except the peak at m/z of 449.15) were among the top 14 peaks in the full data analysis. Three of these peaks were validated as being creatinine, creatine, and 5a-DHT. The remaining peak was ranked 16^th^ (q-value = 0.013) in the full data analysis.

A subgroup analysis was also conducted for the Calgary dataset. This resulted in 8 significant peaks at the Bonferroni threshold of 0.05/2203, with no additional peaks significant at the q-value threshold of 0.01. Six (m/z values of 357.25, 369.17, 114.07, 367.16, 451.17, and 209.12) of these 8 peaks were among the top 14 peaks in the full data analysis, three of them corresponding to the validated metabolites creatinine, 5a-DHT, and either testosterone sulfate, epitestosterone sulfate, or dehydroepiandrosterone sulfate. The remaining 2 peaks at m/z of 223.13 and 175.12 respectively were ranked 15^th^ (q-value = 0.0101), and 612^th^ (q-value > 0.99) in the full data analysis. We note that creatine was only slightly outside of the threshold deemed significant in the Calgary-only analysis (q-value = 0.016).

**Fig 2:**
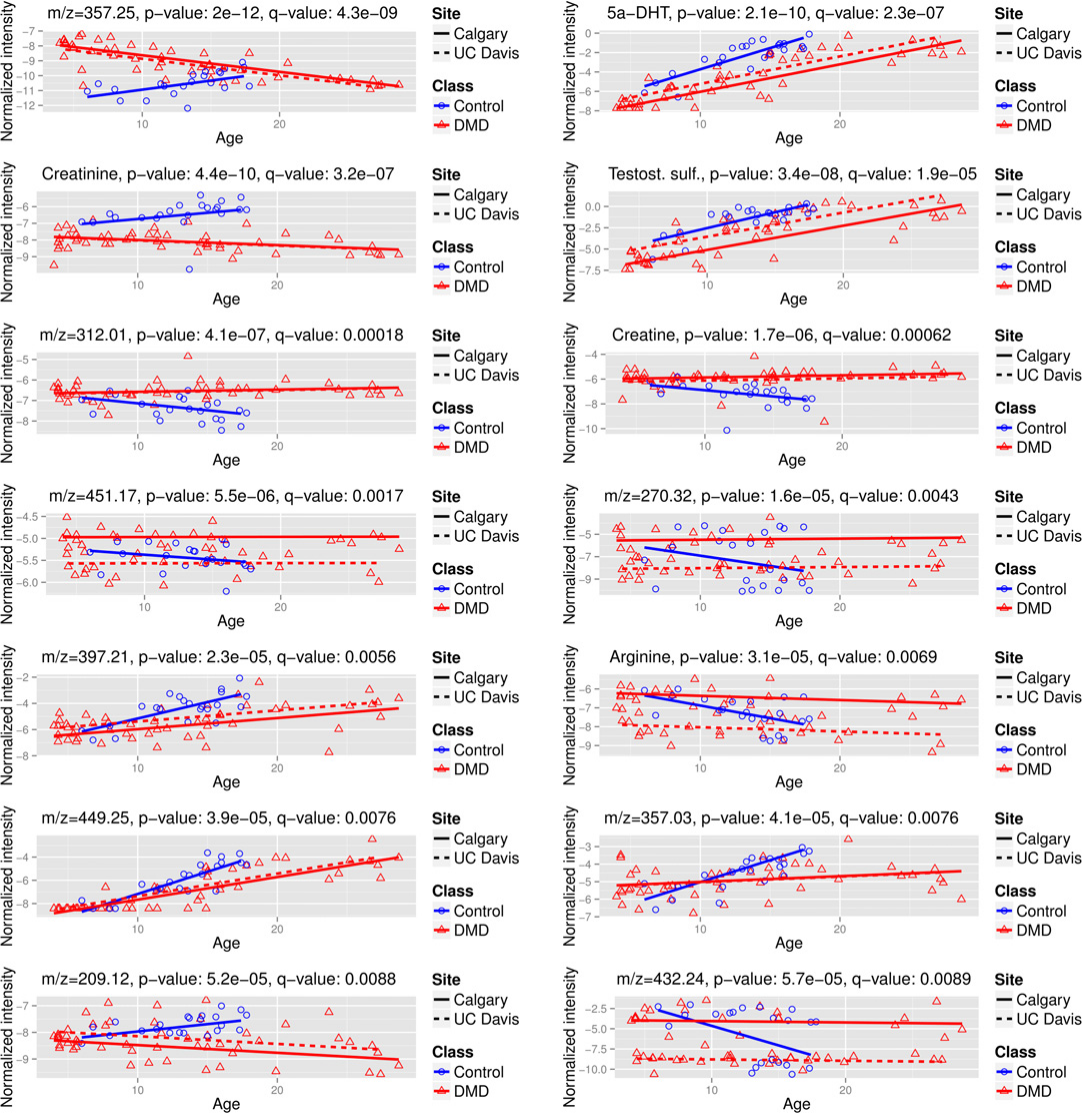
Plots of the intensity versus age for the top 14 metabolites associated with DMD status. The intensity levels have been internal standard normalized, quantile normalized, and log2-transformed. The likely annotations are given for the validated peaks and the m/z value of the adduct in the LC-MS experiment is given for the other peaks; the peak labelled testosterone sulfate may be dehydroepiandrosterone sulfate or epitesterone sulfate. The points are color- and shape-coded by disease status. The regression lines obtained from the interaction model are shown for the Calgary site for DMD cases and controls (solid lines) and for the UC Davis site for DMD cases (broken line).

**Fig 3:**
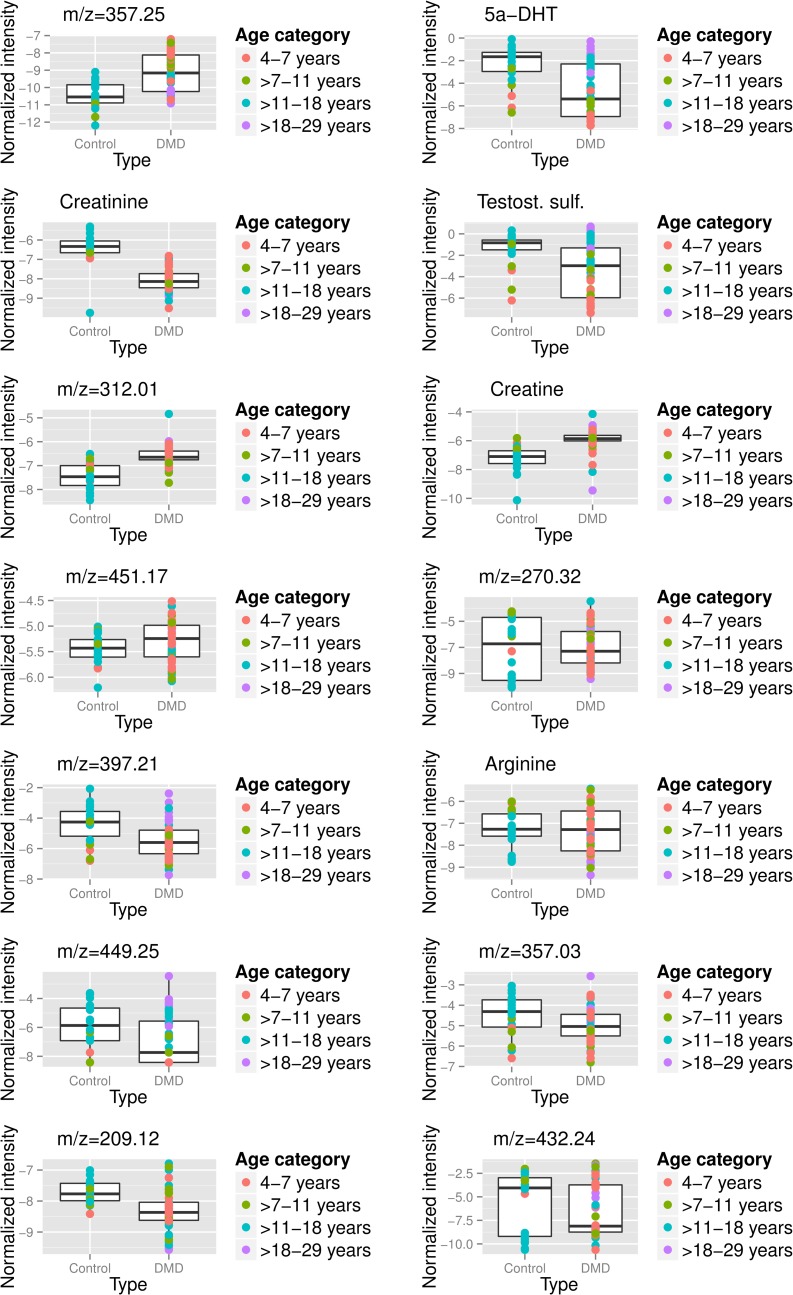
Boxplots of the intensity versus age for the top 14 metabolites associated with DMD status. The intensity levels have been internal standard normalized, quantile normalized, and log2-transformed. The likely annotations are given for the validated peaks and the m/z value of the adduct in the LC-MS experiment is given for the other peaks; the peak labelled testosterone sulfate may be dehydroepiandrosterone sulfate or epitesterone sulfate. The points are separated by DMD status and color-coded by age category.

Fig 2 plots the normalized intensity values versus age for the top 14 peaks, with the regression lines shown for the Calgary and UC Davis sites, which had the largest numbers of participants. It is clearly shown that levels of some metabolites (e.g. creatinine at m/z 114.06) and the unknown compound at m/z = 312) are not substantially different between DMD and healthy volunteers at young ages, then their levels substantially diverge at around 15 years of age. Creatinine was lower in DMD group relative to control group at all ages with the most substantial difference seen at an older age (> 15 years old). Fig 3 displays boxplots for these same metabolites for the two groups and clearly shows substantial differences between DMD and healthy volunteers at older ages (blue dots).

**Fig 4:**
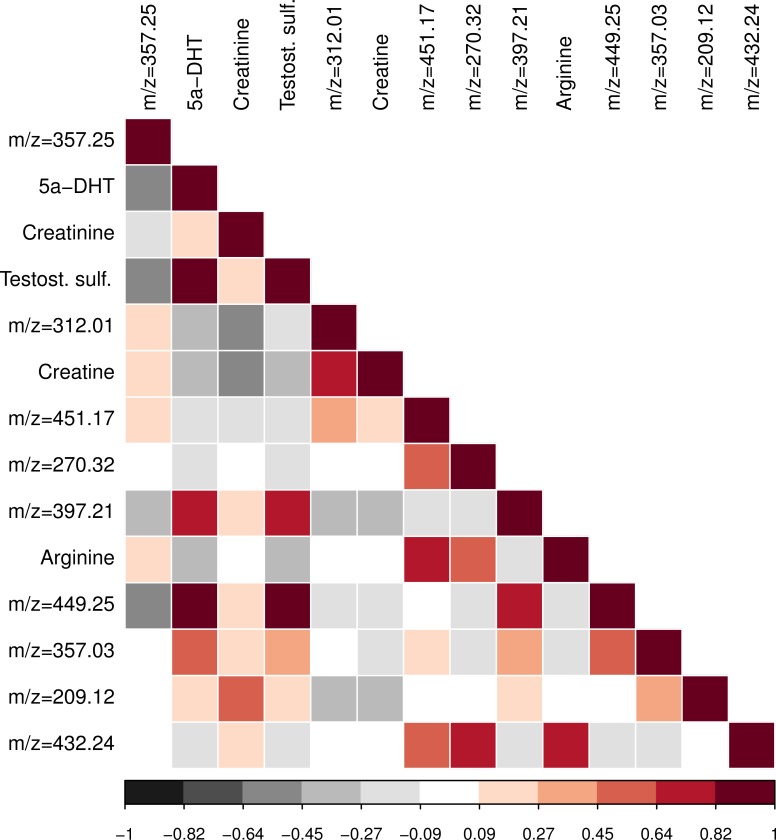
Correlation plot of the top 14 metabolites associated with DMD status. The intensity levels have been internal standard normalized, quantile normalized, and log2-transformed. The likely annotations are given for the validated peaks and the m/z value of the adduct in the LC-MS experiment is given for the other peaks; the peak labelled testosterone sulfate may be dehydroepiandrosterone sulfate or epitesterone sulfate.

The top 14 metabolites include multiple pairs which have strong positive or negative correlations (Fig 4). In particular, we note that creatine and creatinine are negatively correlated (Pearson correlation = -0.48) while the two testosterone metabolites, 5a-DHT and testosterone sulfate (or epitestosterone sulfate or dehydroepiandrosterone sulfate), are strongly positively correlated (Pearson correlation = 0.92).

**Fig 5:**
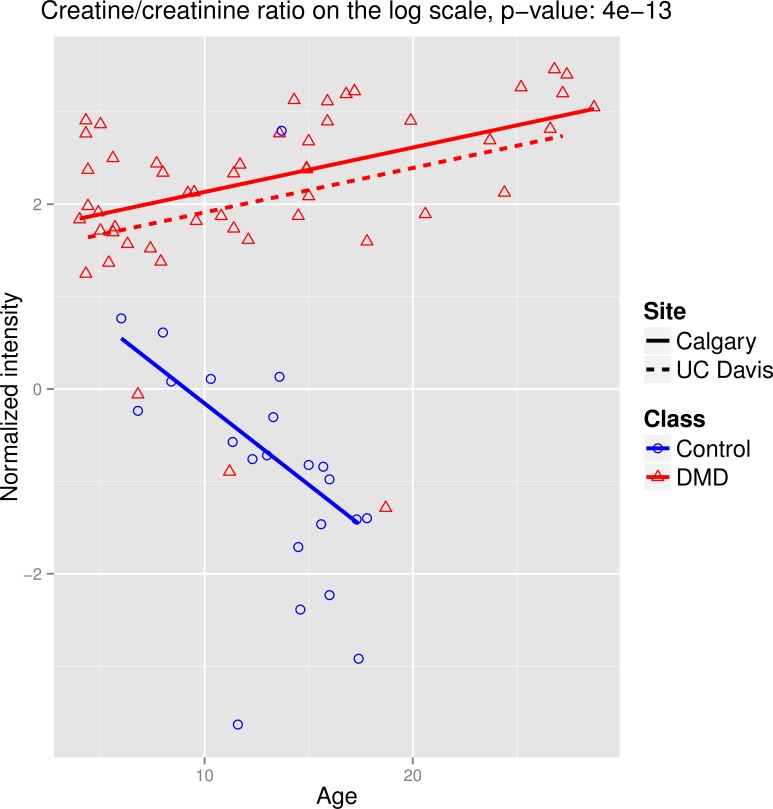
Plot of the creatine/creatinine ratio of intensities on the log_2_ scale versus age. The intensity levels have been internal standard normalized and quantile normalized. The points are color- and shape-coded by disease status. The regression lines obtained from the interaction model are shown for the Calgary site for DMD cases and controls (solid lines) and for the UC Davis site for DMD cases (broken line).

We further considered a similar linear regression analysis for the creatine/creatinine ratio (on the log_2_ scale). Once again an F-test was performed, looking for an effect of disease status in the presence of age and study site and allowing for an interaction between disease status and age; the result was highly significant (p = 4 x 10^−13^). Fig 5 shows the plot of the ratio on the log_2_ scale against participants’ ages. We also compared this ratio to the values of serum CK muscle type (CKM) measured in a recent proteomics study [12]. All 51 of the DMD cases in the current study were also considered in the proteomics study. Overall, there was a negative correlation between CKM level and creatine/creatinine in the cases, reflecting a decrease in CKM activity that is most likely due to loss of muscle mass with age (S2 Fig).

The top 14 metabolites and the creatine/creatinine ratio all had areas under the curve (AUC) values greater than 0.5 in the ROC analysis (S3 Fig) 8 of these 15 biomarkers had AUC greater than 0.8, including creatine, creatinine, creatine/creatinine ratio, and the two testosterone metabolites.

### Pathway analyses for metabolites significantly different between DMD patients and healthy controls

The pathways in which at least one of the 5 validated peaks, corresponding to 7 HMDB IDs–due to the uncertainty for the peak with m/z 367.16 –were considered. Two IDs, corresponding to epitestosterone sulfate and 5-DHT were not found in the ConsensusPathDB database. The pathways in which at least one of the metabolites associated with the remaining 5 HMDB IDs is present are given in S5 Table. Creatine metabolism is the most significant pathway followed by urea cycle and metabolism of amino groups, creatine biosynthesis and arginine and proline metabolism. The validated metabolites present in these pathways are creatine, creatinine, and L-arginine. In addition to the amino acid metabolism pathways, hormonal pathways such as 17-beta hydroxysteroid dehydrogenase III deficiency and androgen and estrogen metabolism were also significant. These pathways contain testosterone sulfate and dehydroepiandrosterone sulfate.

### Bayesian network analyses

Six peaks were identified as important for classification for the full-data analysis, two of them being validated as creatinine and creatine; the remaining 4 peaks were ranked 27^th^, 58^th^, 88^th^, and 1479^th^ using the F-test. Using only these 6 peaks, the participants in the study can be classified at 97% accuracy (in-sample classification). Four of these peaks were identified in at least 4 of the 5 combinations in the five-fold cross-validation, corresponding to creatine (5 times), creatinine (5 times), and the peaks ranked 27^th^ (q-value = 0.04) and 58^th^ (q-value = 0.10), which have m/z values of 154.06 and 176.04, respectively (4 times). The peak with m/z = 154.06 is likely to be annotated to a +Na adduct of creatine or beta-guanidinopropionic acid based on the HMDB database.

The average classification precision for the five-fold validation was 88%.

The Bayesian network analysis applied to the Calgary subset selected only two peaks, corresponding to creatine and creatinine. Using only these two peaks, the samples were classified at 89% accuracy. Five-fold cross validation identified the creatine and creatinine peaks as the only peaks being selected in 3 of the 5 combinations. None of the peaks appeared in more than 3 of the combinations. The total classification precision for the 5 fold subgroup analysis was 71%. The lower classification accuracy of the subgroup analysis may be due to the smaller learning set size.

## Discussion

To the best of our knowledge, this is the first serum metabolomics study of DMD. We considered serum from 51 DMD patients and 22 age-matched controls for discovery of novel metabolic biomarkers for DMD using LC-MS profiling. After data processing, linear regression models were fit for each metabolite, considering the associations with DMD disease status, age, study site, as well as the age-by-DMD status interaction. We found 14 putative molecular biomarkers that differ in their levels between DMD patients and healthy controls when age is taken into consideration. Essentially, as in the DMD proteomics study, we considered increasing age as a surrogate for disease progression among the DMD patients [12]. These metabolites included creatine, creatinine, and testosterone-related steroids, which were confirmed by MS/MS analysis. Creatinine and creatine were, as expected, inversely correlated, with creatinine being generally lower and creatine generally higher in the DMD patients. Creatinine tends to increase with age in the controls and decrease in the DMD patients, while creatine tends to decrease in the controls but stay relatively stable in the DMD patients. Creatinine is a breakdown product from the high energy metabolite phosphocreatine in muscle. With loss of muscle mass in DMD there is a decrease in production of creatinine while creatine that is synthesized by the liver stays at a steady level. These results are also in agreement with a recent study which considers serum creatinine in DMD and the less severe Becker muscular dystrophy phenotype, showing an inverse correlation with a number of measures of disease severity [37], given that in our study creatinine is higher in controls compared to cases and shows a slight decrease in cases with age, which is correlated with disease severity.

We note that the ratio between creatine and creatinine may also serve as a possible biomarker, as it generally appears to increase in DMD patients and decrease in controls (Fig 5) and is more significantly associated with DMD status than either creatine or creatinine individually. It is apparent that DMD patients are not metabolizing creatine as they age, most likely due to loss of muscle mass. These results are also in agreement with the values of serum CKM, which were measured on all the DMD cases in a recent proteomics study [12]. While CKM decreases in DMD cases with age, thus leading to levels more similar to those in healthy controls, which makes it not an ideal marker of disease progression, the creatine to creatinine ratio appears to increase in cases and decrease in controls.

Other interesting biomarkers are testosterone-related steroids that showed an increase with age in both DMD patients and controls. However, they remain lower in the case group compared to the control group. This is most probably due to glucocorticoid use, which is known to cause a decrease in levels of endogenous testosterone and derivatives [38]. We note that we detected two testosterone-related derivatives, 5a-DHT and testosterone sulfate (or epitestosterone sulfate or dehydroepiandrosterone sulfate), which are positively correlated with each other. Our subgroup analyses–one restricted to participants aged 4–18 years and one restricted to the Calgary participants–both confirmed several of the significant peaks from the all-data results. The Bayesian network analysis confirms the importance of creatine and creatinine, although it does not select the testosterone metabolites. We note that our study did not include reliable recording of glucocorticoid use, because this is a natural history study and it has been shown that DMD patients may take either deflazacort or prednisone/prednisolone and that many different dosing regimens exist [39]. This is certainly an important aspect which should be investigated in future work. Additional variables such as muscle mass, dietary intake, disease conditions, and drugs or supplements taken by the study participants at the time of the blood draw are also known to impact metabolism and could not be controlled in the present DMD natural history study, therefore additional studies considering these variable are desirable and could be achieved with a well-controlled cohort in the future.

A major challenge we faced and a common one for this type of study involves separating metabolites related to the disease process from those that are a result of drugs or dietary supplements used by the patients. Several compounds were found to have altered levels in DMD patients compared to controls, but are yet to be identified.

Potential differences between study sites could also be a concern. These were significant for the first principal component, which is not surprising, due to possible differences in demographics and environmental exposures which could not be controlled, although the sample collection, processing and storage protocols were consistent at each site. We note as a limitation of this work the fact that the number of patients in 3 of the five sites was less than 10 and that the second largest site, UC Davis, only provided cases. We adjusted for study site in the linear model and performed the Calgary-only subgroup analysis, which resulted in very similar top hits, to partially address this issue but are aware that in the future more sites should be considered, with a larger number of participants per site and more detailed individual-level demographic and exposure information. A further limitation is due to the retrospective nature of the study–meaning that we cannot tell if the change in a metabolite’s intensity is related to a process which impacts disease progression or is impacted by disease progression. We are presently collecting longitudinal samples within a well-controlled cohort in order to answer these questions and to validate the biomarkers proposed in our current, natural history, study. Our future work will also be powered to detect correlations between clinical outcomes which measure disease severity and metabolic markers. As creatine and creatinine are well-studied metabolites, the use of more targeted methods to measure them as well as their ratio in future studies may also provide additional validation for our results.

## Conclusions

We described herein the first comprehensive metabolomic study for DMD. We believe that this preliminary study represents one of the first steps towards finding metabolic surrogate biomarkers of disease progression in DMD patients. Given that the present work considered a natural history study, important variables could not be controlled. We are hopeful that future work will include prospective longitudinal studies with a larger number of variables collected, as well as more targeted assays, which will provide better insights into temporal trends and causal mechanisms related to DMD progression. While further validation of the results from this initial discovery study is expected to follow from the longitudinal cohort, our current work should provide valuable insights as the first metabolomic signature of DMD and might prove to be a useful reference for future metabolomics studies. Integration with additional types of data–such as genomics, proteomics, and clinical outcomes–is a further avenue of interest.

## Supporting Information

S1 Fig MS/MS spectra for the top peaks. (PDF)

S2 Fig Plot of CKM versus the creatine/creatinine ratio in DMD cases, both on the log_2_ scale. The intensity levels for creatine and creatinine have been internal standard normalized and quantile normalized. The points are color- and shape-coded by age category. ρ represents the correlation on the log scale. Both the overall correlation and the correlations within age categories are given.
(PDF)

S3 Fig ROC curves for the top 14 peaks from the overall analysis and for the creatine/creatinine ratio. A logistic model was fit with case/control status as outcome and age and peak intensity as explanatory variables. The AUC for each possible biomarker is given in the title; tpr = true positive rate, fpr = false positive rate.
(PDF)

S1 Table Individual characteristics and peak intensities of the study participants. Each peak is labelled according to its m/z value, retention time in seconds, and mode (‘p’ = positive, ‘n’ = negative); for example, peak M132T37p has an m/z value of 132, a retention time of 37 seconds, and was detected in the positive mode. The log_2_-transformed CKM values from the [12] study are also given for the common samples.
(XLSX)

S2 Table Models considered for PC1. The 9 linear models considered for PC1. The variables and Akaike Information Criteria (AIC) are given. Smaller AIC values indicate a better model fit, while also adjusting for the number of parameters, with the best model according to the AIC being shown in bold. The relative probabilities representing the strength of evidence for each model *j* compared to the model deemed to be the best are calculated by exp(-(AIC_j_-AIC_min_)/2); for instance, the model including only site has 0.43 times as much evidence than the model including both age and site.
(XLSX)

S3 Table Annotations for the top peaks detected in positive mode from HMDB and Metlin. Drugs and non-human metabolites are shown in red. The most likely annotations are highlighted.
(XLSX)

S4 Table Annotations for the top peaks detected in negative mode from HMDB and Metlin. Drugs and non-human metabolites are shown in red. The most likely annotations are highlighted.
(XLSX)

S5 Table Pathways from ConsensusPathDB in which at least one of the 5 validated peaks is present. The ‘Size’ column represents the absolute size of the pathway, the ‘Effective size’ column represents the number of set members that are annotated with an ID of the user-specified ID type (HMDB ID in this case); the ‘Input overlap’ column represents number of entities from the user input list that overlap with entities in the pathway based set. The p-values are obtained from a hypergeometric test of over-representation. The q-values are calculated using the Benjamini-Hochberg approach considering just the tests for the pathways which include at least one validated peak.
(XLSX)

## References

[1] Danieli G, Mostacciuolo M, Bonfante A, Angelini C. Duchenne muscular dystrophy. Human genetics. 1977;35(2):225–31. 844870 10.1007/BF00393974

[2] Emery AE. Population frequencies of inherited neuromuscular diseases—a world survey. Neuromuscular disorders. 1991;1(1):19–29. 1822774 10.1016/0960-8966(91)90039-u

[3] Mah JK, Korngut L, Dykeman J, Day L, Pringsheim T, Jette N. A systematic review and meta-analysis on the epidemiology of Duchenne and Becker muscular dystrophy. Neuromuscular Disorders. 2014;24(6):482–91. 10.1016/j.nmd.2014.03.008 24780148

[4] Bushby K, Finkel R, Birnkrant DJ, Case LE, Clemens PR, Cripe L, et al DMD Care Considerations Working Group: Diagnosis and management of Duchenne muscular dystrophy, part 1: diagnosis, and pharmacological and psychosocial management. Lancet Neurol. 2010;9(1):77–93. 10.1016/S1474-4422(09)70271-6 19945913

[5] McDonald CM, Henricson EK, Abresch RT, Han JJ, Escolar DM, Florence JM, et al The cooperative international neuromuscular research group Duchenne natural history study—a longitudinal investigation in the era of glucocorticoid therapy: design of protocol and the methods used. Muscle & nerve. 2013;48(1):32–54. 23677550 10.1002/mus.23807 PMC4147958

[6] Hoffman EP, Brown RH Jr, Kunkel LM. Dystrophin: the protein product of the Duchenne muscular dystrophy locus. Cell. 1987;51(6):919–28. 3319190 10.1016/0092-8674(87)90579-4

[7] McAdam LC, Mayo AL, Alman BA, Biggar WD. The Canadian experience with long term deflazacort treatment in Duchenne muscular dystrophy. Acta Myologica. 2012;31(1):16 22655512 PMC3440807

[8] Henricson EK, Abresch RT, Cnaan A, Hu F, Duong T, Arrieta A, et al The cooperative international neuromuscular research group Duchenne natural history study: glucocorticoid treatment preserves clinically meaningful functional milestones and reduces rate of disease progression as measured by manual muscle testing and other commonly used clinical trial outcome measures. Muscle & nerve. 2013;48(1):55–67. 23649481 10.1002/mus.23808 PMC4103170

[9] Hoffman EP, Connor EM. Orphan drug development in muscular dystrophy: update on two large clinical trials of dystrophin rescue therapies. Discovery medicine. 2013;16(89):233–9. 24229740

[10] Okinaka S, Kumagai H, Ebashi S, Sugita H, Momoi H, Toyokura Y, et al Serum creatine phosphokinase: Activity in progressive muscular dystrophy and neuromuscular diseases. Archives of neurology. 1961;4(5):520–5. 13730599 10.1001/archneur.1961.00450110050006

[11] Plauchu H, Dellamonica C, Pascal B, Jolivet M, Guibaud P, Cotte J, et al [Neonatal screening for duchenne myopathy by serum elevation of creatine phosphokinase activity. 5 years experience]. Journal de genetique humaine. 1981;29(1):59–69. 7334342

[12] Hathout Y, Brody E, Clemens PR, Cripe L, DeLisle RK, Furlong P, et al Large-scale serum protein biomarker discovery in Duchenne muscular dystrophy. Proceedings of the National Academy of Sciences. 2015:201507719. 10.1073/pnas.1507719112 PMC4466703 26039989

[13] Eisenberg I, Eran A, Nishino I, Moggio M, Lamperti C, Amato AA, et al Distinctive patterns of microRNA expression in primary muscular disorders. Proceedings of the National Academy of Sciences. 2007;104(43):17016–21. 10.1073/pnas.0708115104 PMC2040449 17942673

[14] Cacchiarelli D, Legnini I, Martone J, Cazzella V, d'Amico A, Bertini E, et al miRNAs as serum biomarkers for Duchenne muscular dystrophy. EMBO molecular medicine. 2011;3(5):258–65. 10.1002/emmm.201100133 21425469 PMC3112257

[15] Zaharieva IT, Calissano M, Scoto M, Preston M, Cirak S, Feng L, et al Dystromirs as serum biomarkers for monitoring the disease severity in Duchenne muscular dystrophy. PloS one. 2013;8(11):e80263 10.1371/journal.pone.0080263 24282529 PMC3840009

[16] Nadarajah V, van Putten M, Chaouch A, Garrood P, Straub V, Lochmüller H, et al Serum matrix metalloproteinase-9 (MMP-9) as a biomarker for monitoring disease progression in Duchenne muscular dystrophy (DMD). Neuromuscular Disorders. 2011;21(8):569–78. 10.1016/j.nmd.2011.05.011 21724396

[17] Cynthia Martin F, Hiller M, Spitali P, Oonk S, Dalebout H, Palmblad M, et al Fibronectin is a serum biomarker for Duchenne muscular dystrophy. PROTEOMICS-Clinical Applications. 2014;8(3–4):269–78. 10.1002/prca.201300072 24458521

[18] Hathout Y, Marathi RL, Rayavarapu S, Zhang A, Brown KJ, Seol H, et al Discovery of serum protein biomarkers in the mdx mouse model and cross-species comparison to Duchenne muscular dystrophy patients. Human molecular genetics. 2014:ddu366. 10.1093/hmg/ddu366 PMC4240201 25027324

[19] Rouillon J, Zocevic A, Leger T, Garcia C, Camadro J-M, Udd B, et al Proteomics profiling of urine reveals specific titin fragments as biomarkers of Duchenne muscular dystrophy. Neuromuscular Disorders. 2014;24(7):563–73. 10.1016/j.nmd.2014.03.012 24813925

[20] Ayoglu B, Chaouch A, Lochmüller H, Politano L, Bertini E, Spitali P, et al Affinity proteomics within rare diseases: a BIO‐NMD study for blood biomarkers of muscular dystrophies. EMBO molecular medicine. 2014;6(7):918–36. 10.15252/emmm.201303724 24920607 PMC4119355

[21] Griffin JL, Des Rosiers C. Applications of metabolomics and proteomics to the mdx mouse model of Duchenne muscular dystrophy: lessons from downstream of the transcriptome. Genome Med. 2009;1(3):32 10.1186/gm32 19341503 PMC2664943

[22] Wishart DS, Jewison T, Guo AC, Wilson M, Knox C, Liu Y, et al HMDB 3.0—the human metabolome database in 2013. Nucleic acids research. 2012:gks1065. 10.1093/nar/gks1065 PMC3531200 23161693

[23] Wang TJ, Larson MG, Vasan RS, Cheng S, Rhee EP, McCabe E, et al Metabolite profiles and the risk of developing diabetes. Nat Med. 2011;17(4):448–53. 10.1038/nm.2307 21423183 PMC3126616

[24] Suhre K, Meisinger C, Doring A, Altmaier E, Belcredi P, Gieger C, et al Metabolic footprint of diabetes: a multiplatform metabolomics study in an epidemiological setting. PLoS One. 2010;5(11):e13953 10.1371/journal.pone.0013953 21085649 PMC2978704

[25] Abate-Shen C, Shen MM. Diagnostics: The prostate-cancer metabolome. Nature. 2009;457(7231):799–800. 10.1038/457799a . 19212391

[26] Jansson J, Willing B, Lucio M, Fekete A, Dicksved J, Halfvarson J, et al Metabolomics reveals metabolic biomarkers of Crohn's disease. PLoS One. 2009;4(7):e6386 10.1371/journal.pone.0006386 19636438 PMC2713417

[27] Smith CA, Want EJ, O'Maille G, Abagyan R, Siuzdak G. XCMS: processing mass spectrometry data for metabolite profiling using nonlinear peak alignment, matching, and identification. Analytical chemistry. 2006;78(3):779–87. 16448051 10.1021/ac051437y

[28] Kuhl C, Tautenhahn R, Böttcher C, Larson TR, Neumann S. CAMERA: an integrated strategy for compound spectra extraction and annotation of liquid chromatography/mass spectrometry data sets. Analytical chemistry. 2011;84(1):283–9. 10.1021/ac202450g 22111785 PMC3658281

[29] Guertin KA, Moore SC, Sampson JN, Huang W-Y, Xiao Q, Stolzenberg-Solomon RZ, et al Metabolomics in nutritional epidemiology: identifying metabolites associated with diet and quantifying their potential to uncover diet-disease relations in populations. The American journal of clinical nutrition. 2014:ajcn. 078758. 10.3945/ajcn.113.078758 PMC4144099 24740205

[30] Leek JT, Scharpf RB, Bravo HC, Simcha D, Langmead B, Johnson WE, et al Tackling the widespread and critical impact of batch effects in high-throughput data. Nature Reviews Genetics. 2010;11(10):733–9. 10.1038/nrg2825 20838408 PMC3880143

[31] Benjamini Y, Hochberg Y. Controlling the false discovery rate: a practical and powerful approach to multiple testing. Journal of the Royal Statistical Society Series B (Methodological). 1995:289–300.

[32] Storey JD. A direct approach to false discovery rates. Journal of the Royal Statistical Society: Series B (Statistical Methodology). 2002;64(3):479–98.

[33] Smith CA, O'Maille G, Want EJ, Qin C, Trauger SA, Brandon TR, et al METLIN: a metabolite mass spectral database. Therapeutic drug monitoring. 2005;27(6):747–51. 16404815 10.1097/01.ftd.0000179845.53213.39

[34] Kamburov A, Stelzl U, Lehrach H, Herwig R. The ConsensusPathDB interaction database: 2013 update. Nucleic acids research. 2013;41(D1):D793–D800. 23143270 10.1093/nar/gks1055 PMC3531102

[35] Pearl J. Probabilistic reasoning in intelligent systems: networks of plausible inference: Morgan Kaufmann; 1988.

[36] Burnham KP, Anderson DR, Huyvaert KP. AIC model selection and multimodel inference in behavioral ecology: some background, observations, and comparisons. Behavioral Ecology and Sociobiology. 2011;65(1):23–35.

[37] Zhang H, Zhu Y, Sun Y, Liang Y, Li Y, Zhang Y, et al Serum Creatinine Level: A Supplemental Index to Distinguish Duchenne Muscular Dystrophy from Becker Muscular Dystrophy. Disease markers. 2015. 10.1155/2015/141856 PMC4380093 25852218

[38] Morrison D, Capewell S, Reynolds S, Thomas J, Ali N, Read G, et al Testosterone levels during systemic and inhaled corticosteroid therapy. Respiratory medicine. 1994;88(9):659–63. 7809437 10.1016/s0954-6111(05)80062-9

[39] Bello L, Gordish-Dressman H, Morgenroth L, Henricson E, Duong T, Hoffman E, et al Prednisone/prednisolone and deflazacort differ in long term outcomes on ambulation and side effects in the CINRG Duchenne Natural History Study (S50. 001). Neurology. 2015;84(14 Supplement):S50. 001. 10.1212/WNL.0000000000001950 PMC4603595 26311750

