# Correction: Discovery of Metabolic Biomarkers for Duchenne Muscular Dystrophy within a Natural History Study

**DOI:** 10.1371/journal.pone.0159895

**Published:** 2016-07-19

**Authors:** Simina M. Boca, Maki Nishida, Michael Harris, Shruti Rao, Amrita K. Cheema, Kirandeep Gill, Difei Wang, Lin An, Robinder Gauba, Haeri Seol, Lauren P. Morgenroth, Erik Henricson, Craig McDonald, Jean K. Mah, Paula R. Clemens, Eric P. Hoffman, Yetrib Hathout, Subha Madhavan

Drs. Difei Wang, Lin An, and Robinder Gauba are not included in the author byline. Please view the corrected author byline here:

Simina M. Boca^1,2,3^, Maki Nishida^1^, Michael Harris^1^, Shruti Rao^1^, Amrita K. Cheema^2,4^, Kirandeep Gill^2^, Difei Wang^1,2^, Lin An^1^, Robinder Gauba^1^, Haeri Seol^5^, Lauren P. Morgenroth^5^, Erik Henricson^6^, Craig McDonald^6^, Jean K. Mah^7^, Paula R. Clemens^8,9^, Eric P. Hoffman^5^, Yetrib Hathout^5^, Subha Madhavan^1,2^

1 Innovation Center for Biomedical Informatics, Georgetown University Medical Center, Washington, DC, United States of America,

2 Department of Oncology, Georgetown University Medical Center, Washington, DC, United States of America,

3 Department of Biostatistics, Bioinformatics, and Biomathematics, Georgetown University Medical Center, Washington, DC, United States of America,

4 Department of Biochemistry and Molecular & Cellular Biology, Georgetown University Medical Center, Washington, DC, United States of America,

5 Children’s National Medical Center and the George Washington University, Washington, DC, United States of America,

6 Department of Physical Medicine and Rehabilitation, University of California Davis, School of Medicine, Davis, California, United States of America,

7 Department of Pediatrics, University of Calgary, Alberta Children’s Hospital, Calgary, Alberta, Canada,

8 Neurology Service, Department of Veteran Affairs Medical Center, Pittsburgh, Pennsylvania, United States of America,

9 Department of Neurology, University of Pittsburgh, Pittsburgh, Pennsylvania, United States of America

The contributions of Dr. Wang, Dr. An, and Dr. Gauba are as follows: Contributed reagents/materials/analysis tools.
