# Supplementary material for: Discovery of Metabolic Biomarkers for Duchenne Muscular Dystrophy within a Natural History Study
Source: PLoS One. 2016 Apr 15;11(4):e0153461. doi: 10.1371/journal.pone.0153461 (PMC4833348; doi:10.1371/journal.pone.0153461)
Supplement: S1 Fig — (PDF) [file pone.0153461.s001.pdf]

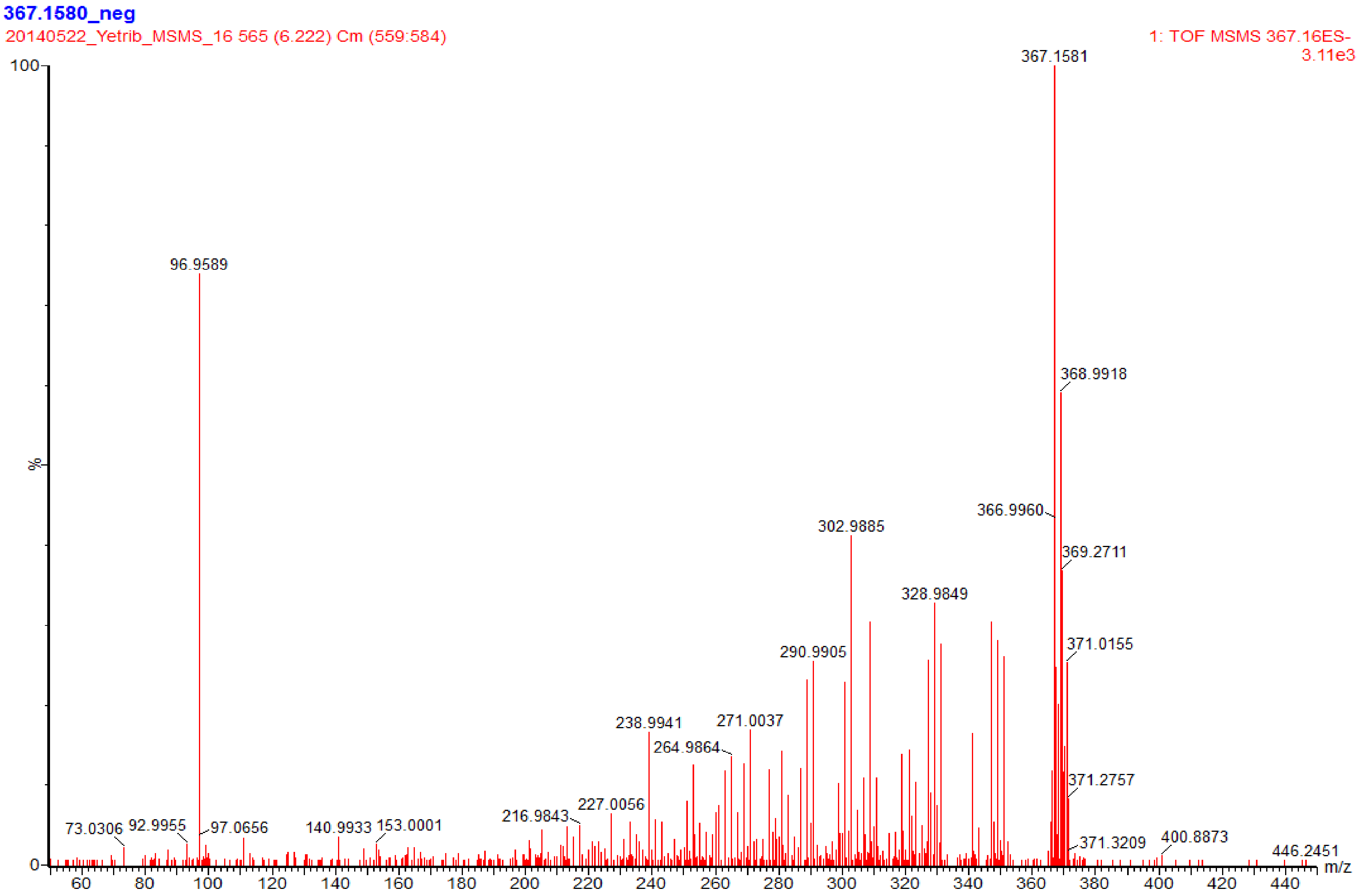
367.1580_neg
20140522_Yetrib_MSMS_16 565 (6.222) Cm (559:584)
1: TOF MSMS 367.16ES-
3.11e3
367.1581
96.9589
368.9918
366.9960
302.9885
369.2711
328.9849
290.9905
371.0155
238.9941
271.0037
264.9864
371.2757
227.0056
216.9843
73.0306
92.9955
97.0656
140.9933
153.0001
371.3209
400.8873
446.2451
100
%
0
m/z
60
80
100
120
140
160
180
200
220
240
260
280
300
320
340
360
380
400
420
440

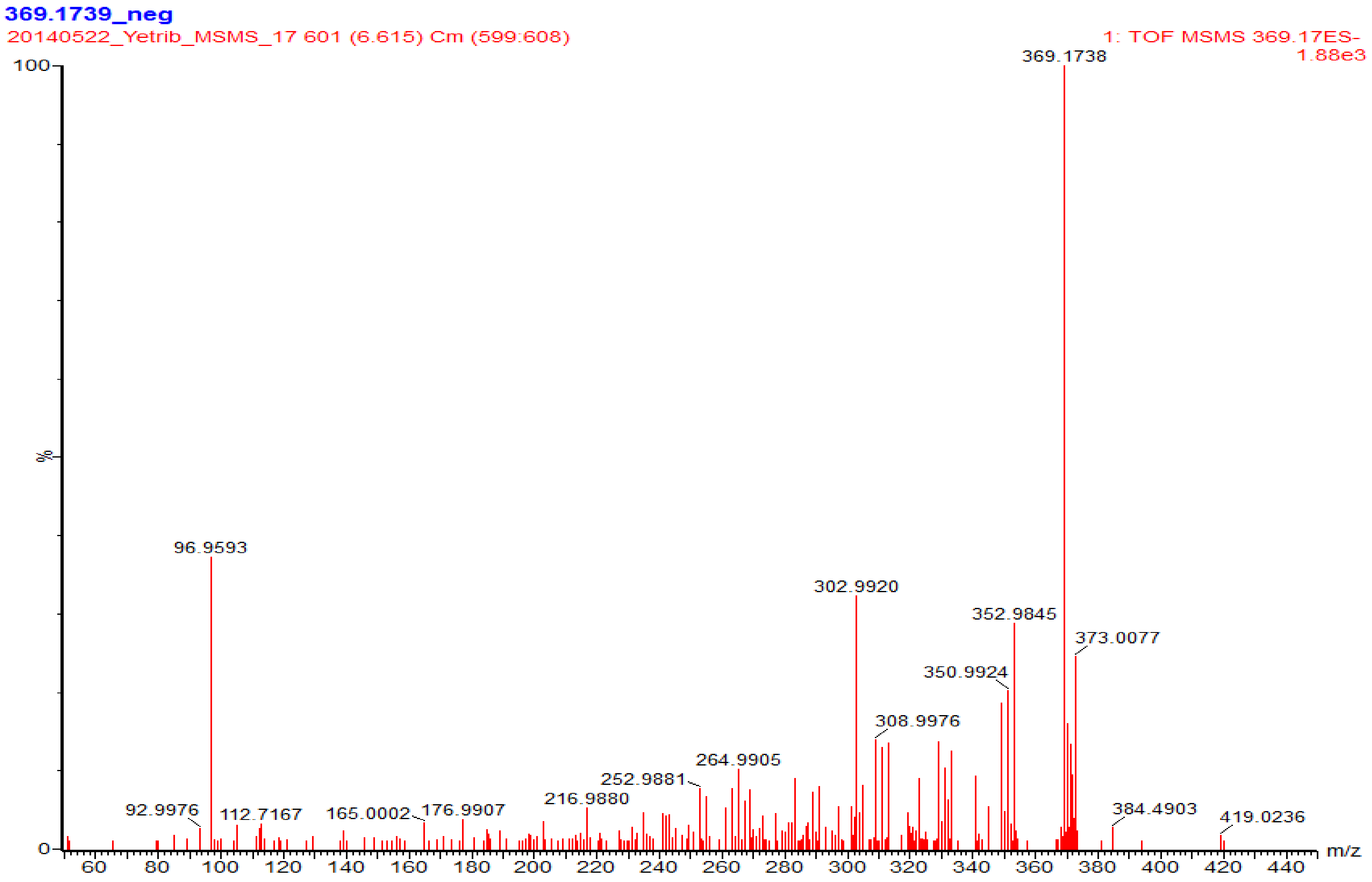

369.1739_neg
20140522_Yetrib_MSMS_17 601 (6.615) Cm (599:608)
1: TOF MSMS 369.17ES-
1.88e3
369.1738
96.9593
302.9920
352.9845
373.0077
350.9924
308.9976
264.9905
252.9881
216.9880
92.9976
112.7167
165.0002
176.9907
384.4903
419.0236
100
%
0
m/z
60
80
100
120
140
160
180
200
220
240
260
280
300
320
340
360
380
400
420
440

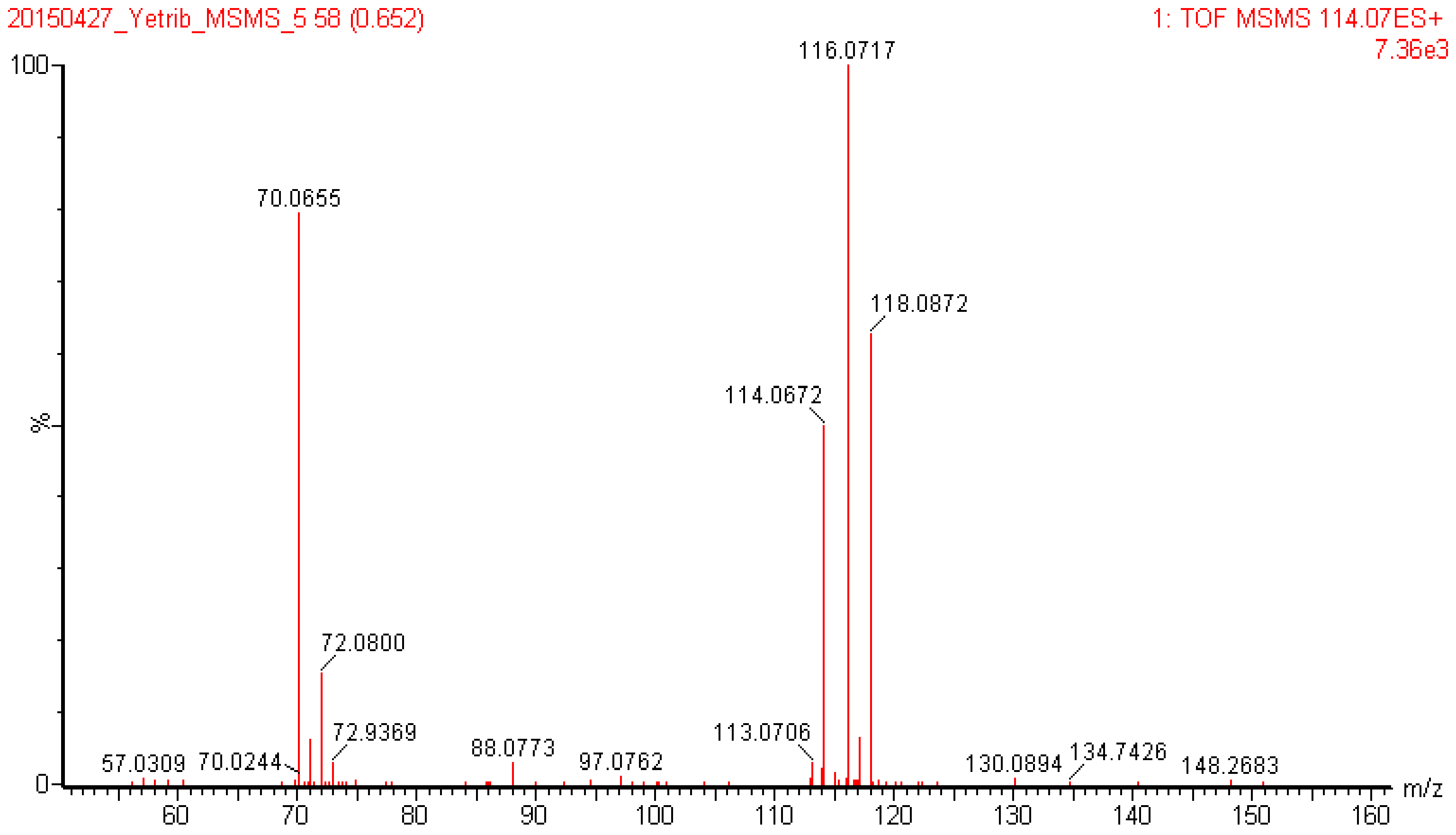
20150427_Yetrib_MSMS_5 58 (0.652)
1: TOF MSMS 114.07ES+
7.36e3
116.0717
70.0655
118.0872
114.0672
72.0800
72.9369
88.0773
113.0706
57.0309
70.0244
97.0762
130.0894
134.7426
148.2683
100
%
0
60
70
80
90
100
110
120
130
140
150
160
m/z

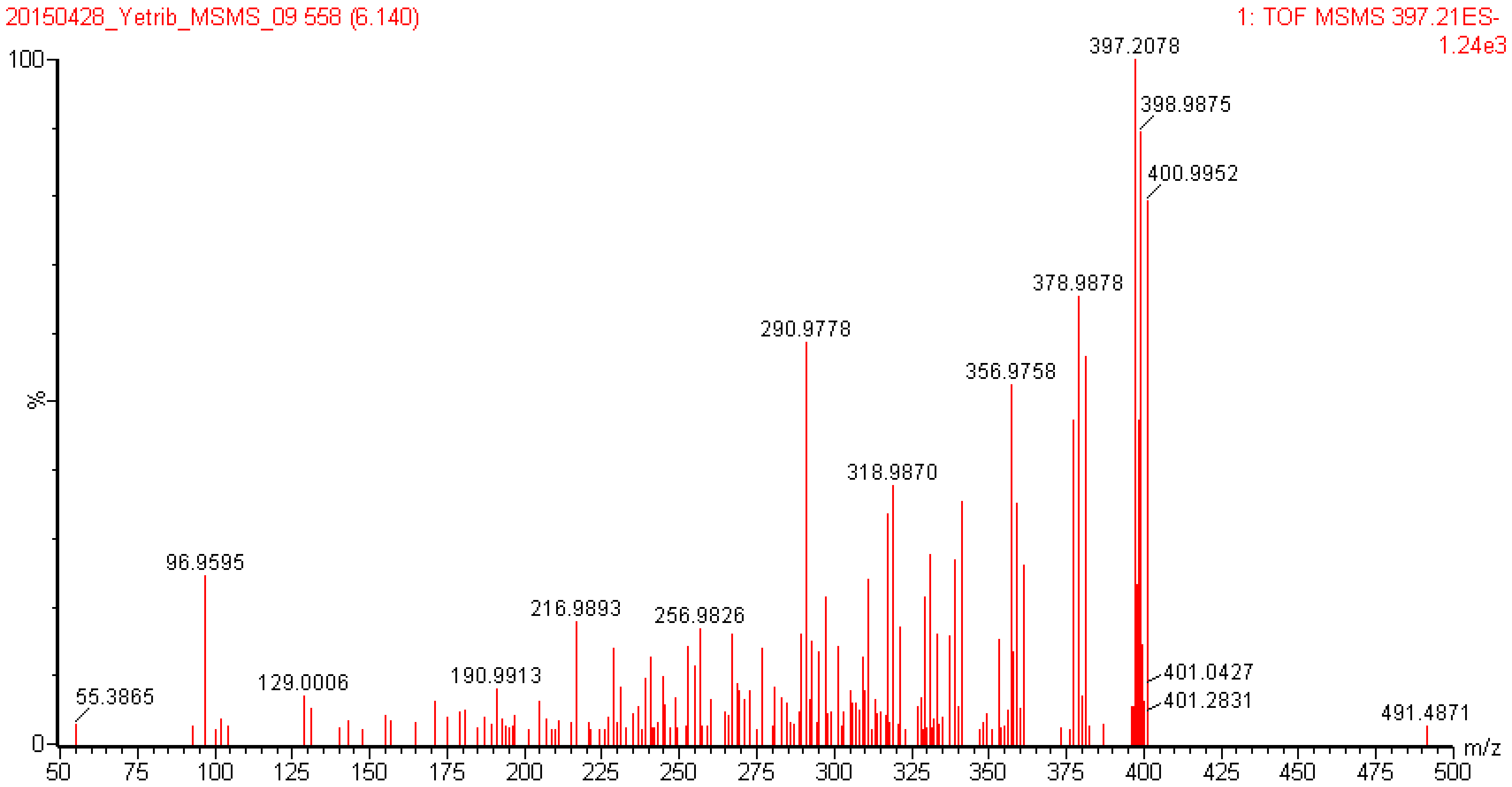
20150428_Yetrib_MSMS_09 558 (6.140)
1: TOF MSMS 397.21ES-
1.24e3
397.2078
398.9875
400.9952
378.9878
290.9778
356.9758
318.9870
96.9595
216.9893
256.9826
401.0427
401.2831
190.9913
129.0006
55.3865
491.4871
100
%
0
m/z
50
75
100
125
150
175
200
225
250
275
300
325
350
375
400
425
450
475
500

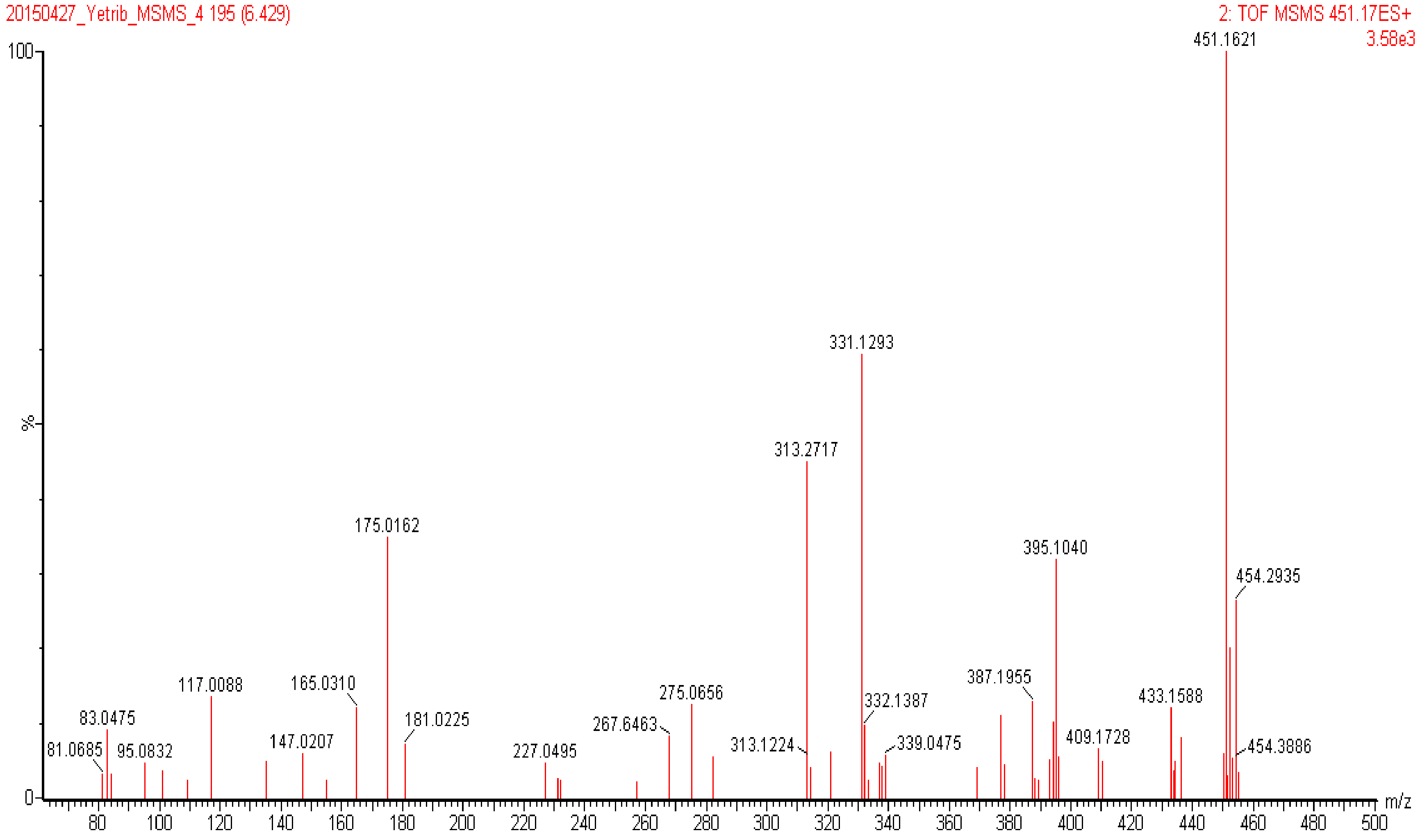

20150427_Yetrib_MSMS_4 195 (6.429)
2: TOF MSMS 451.17ES+
3.58e3
451.1621
331.1293
313.2717
175.0162
395.1040
454.2935
117.0088
165.0310
83.0475
181.0225
81.0685
95.0832
147.0207
227.0495
267.6463
275.0656
313.1224
332.1387
339.0475
387.1955
409.1728
433.1588
454.3886
100
%
0
m/z
80
100
120
140
160
180
200
220
240
260
280
300
320
340
360
380
400
420
440
460
480
500

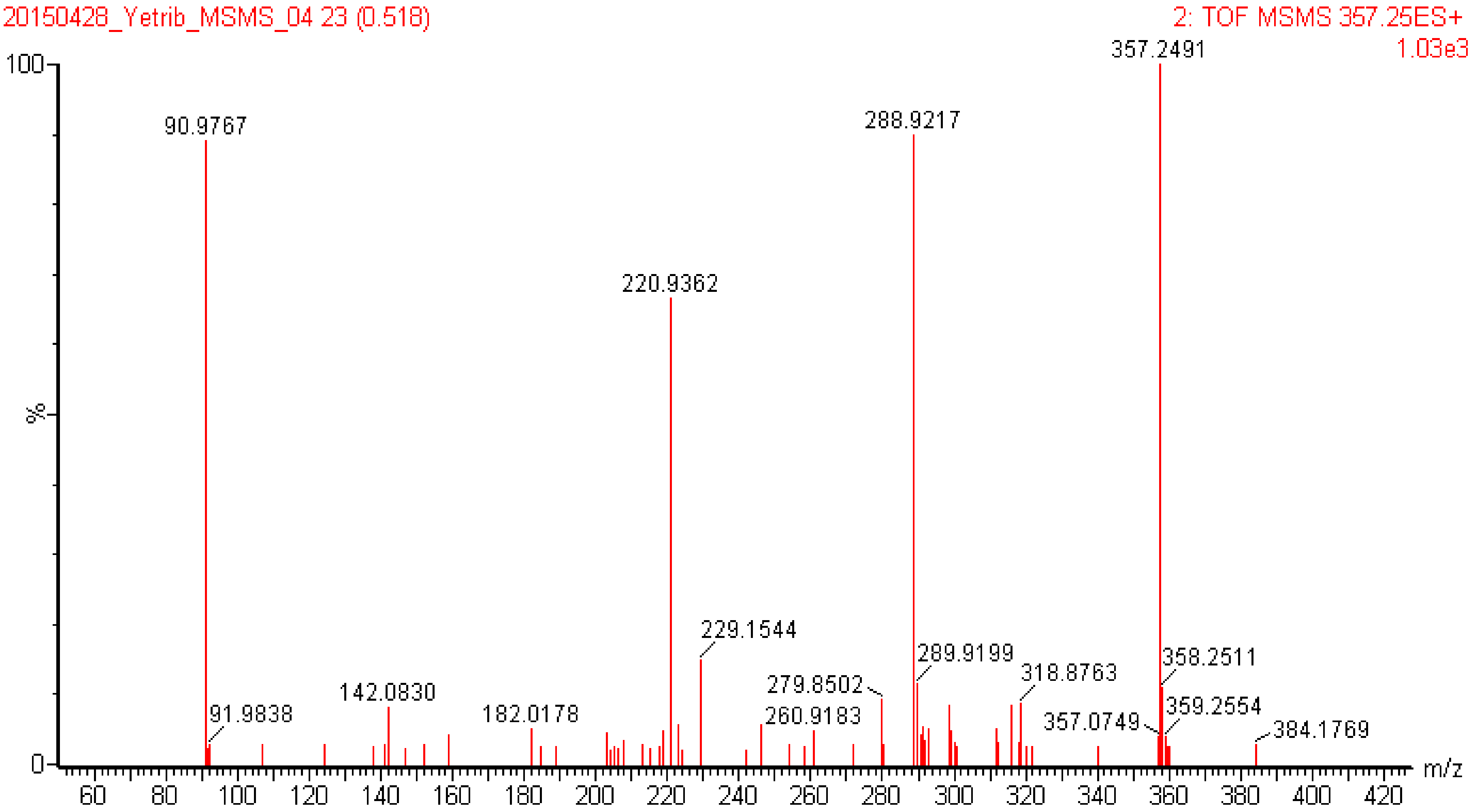
20150428_Yetrib_MSMS_04 23 (0.518)
2: TOF MSMS 357.25ES+
1.03e3
357.2491
90.9767
288.9217
220.9362
229.1544
289.9199
358.2511
318.8763
279.8502
359.2554
260.9183
357.0749
384.1769
142.0830
91.9838
182.0178
100
%
0
60
80
100
120
140
160
180
200
220
240
260
280
300
320
340
360
380
400
420
m/z

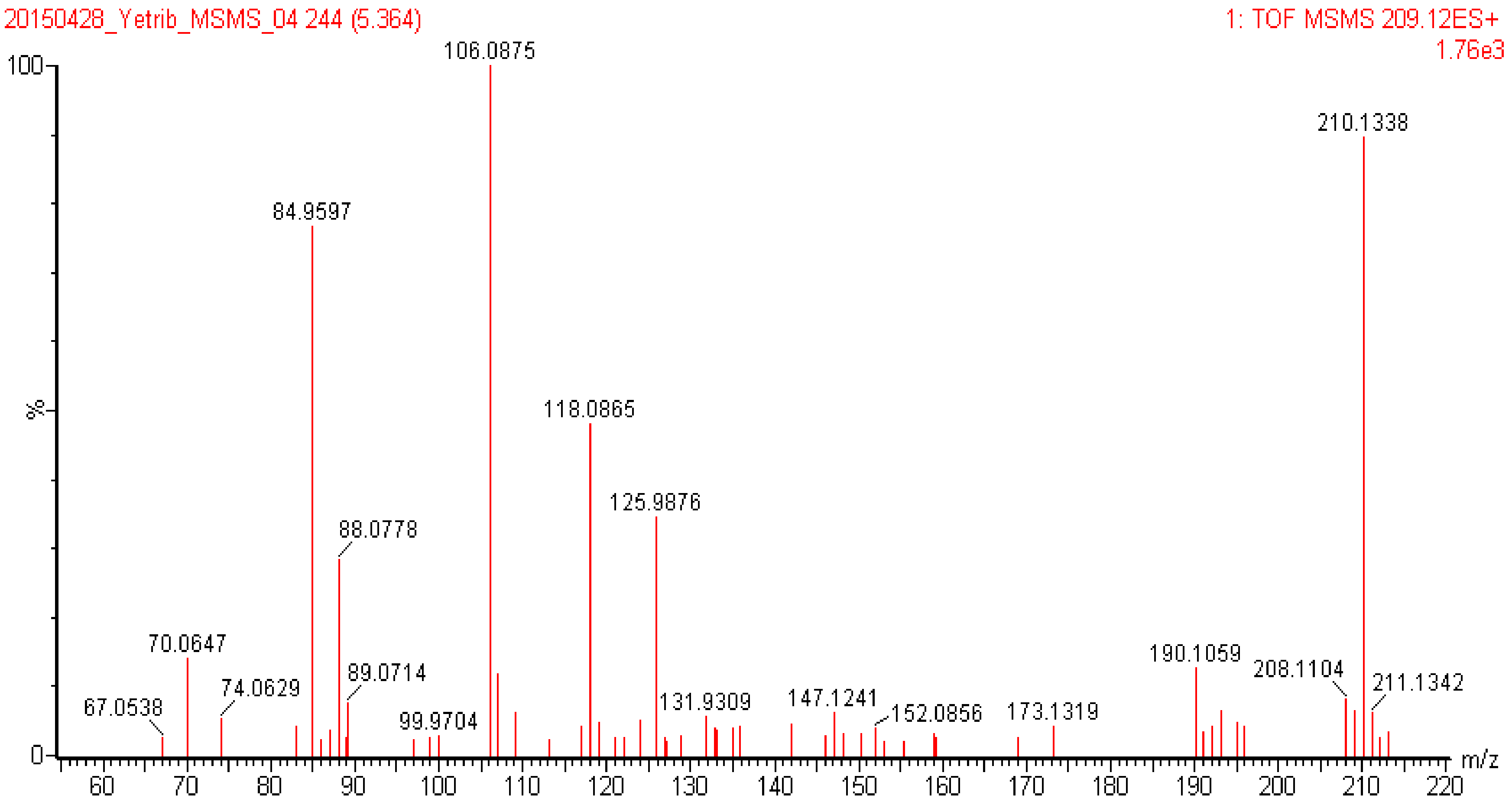

20150428_Yetrib_MSMS_04 244 (5.364)
1: TOF MSMS 209.12ES+
1.76e3
100
%
0
106.0875
210.1338
84.9597
118.0865
125.9876
88.0778
70.0647
190.1059
208.1104
211.1342
89.0714
74.0629
67.0538
131.9309
147.1241
152.0856
173.1319
99.9704
m/z
60
70
80
90
100
110
120
130
140
150
160
170
180
190
200
210
220

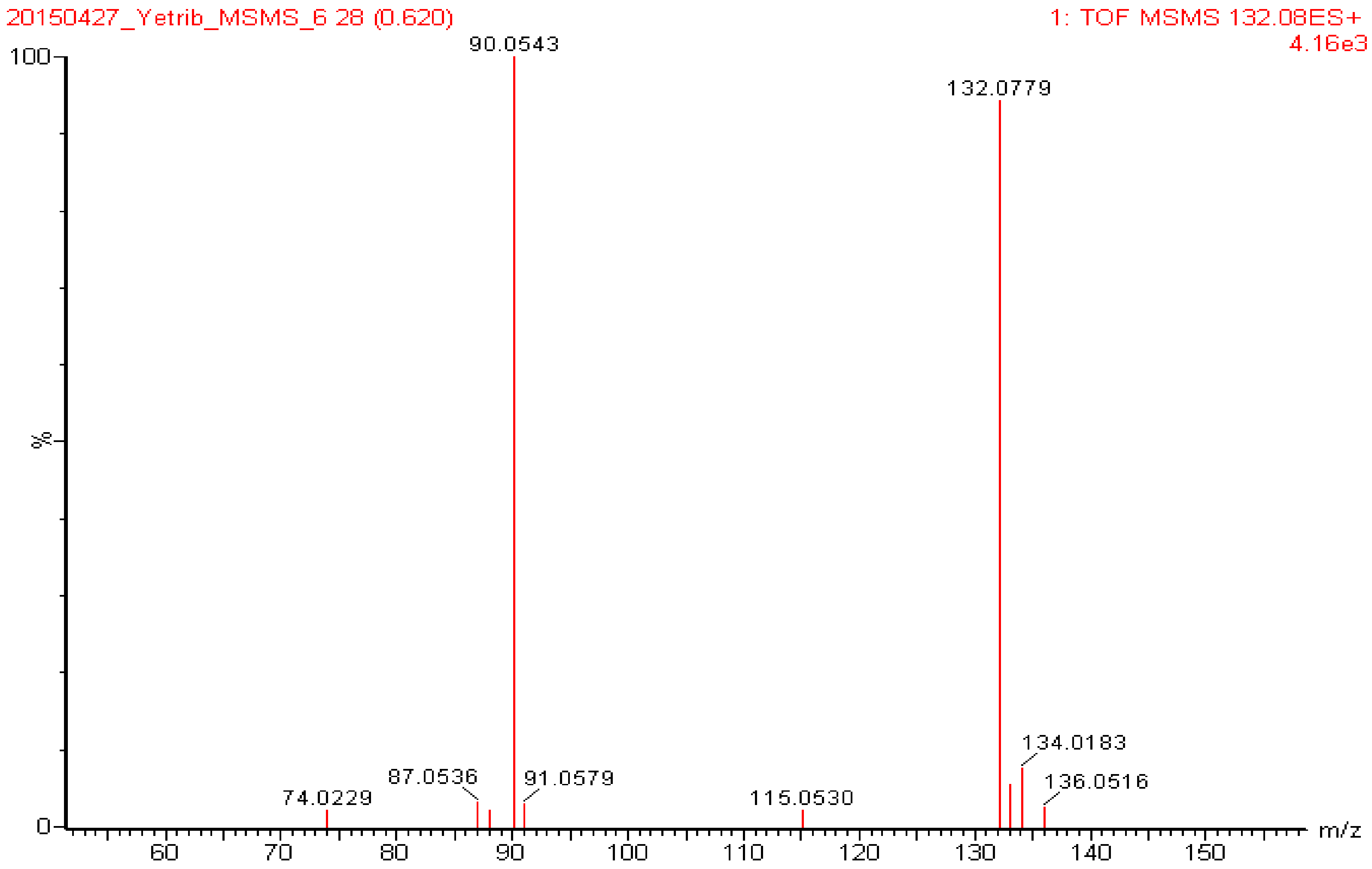
20150427_Yetrib_MSMS_6 28 (0.620)
1: TOF MSMS 132.08ES+
4.16e3
100
%
0
90.0543
132.0779
74.0229
87.0536
91.0579
115.0530
134.0183
136.0516
60
70
80
90
100
110
120
130
140
150
m/z

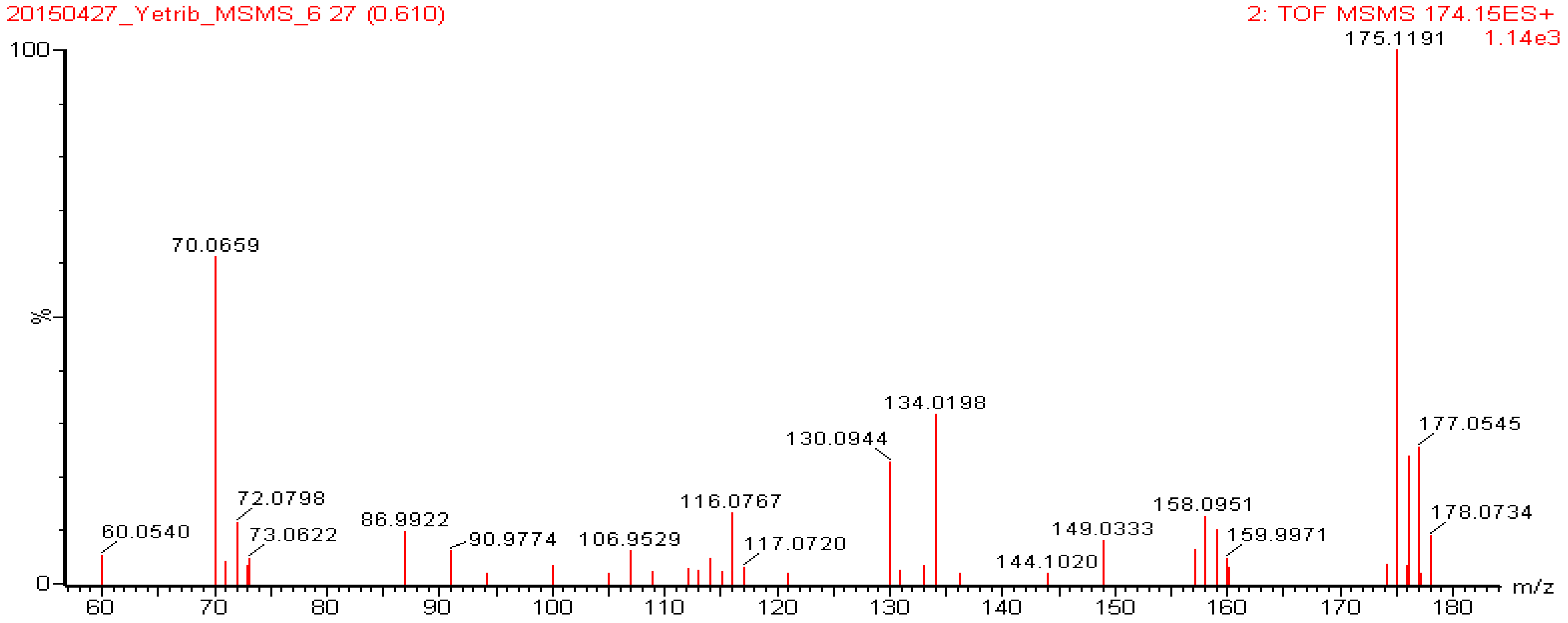
20150427_Yetrib_MSMS_6 27 (0.610)
2: TOF MSMS 174.15ES+
1.14e3
175.1191
70.0659
134.0198
130.0944
177.0545
178.0734
72.0798
116.0767
158.0951
60.0540
86.9922
149.0333
73.0622
90.9774
106.9529
117.0720
159.9971
144.1020
%
m/z

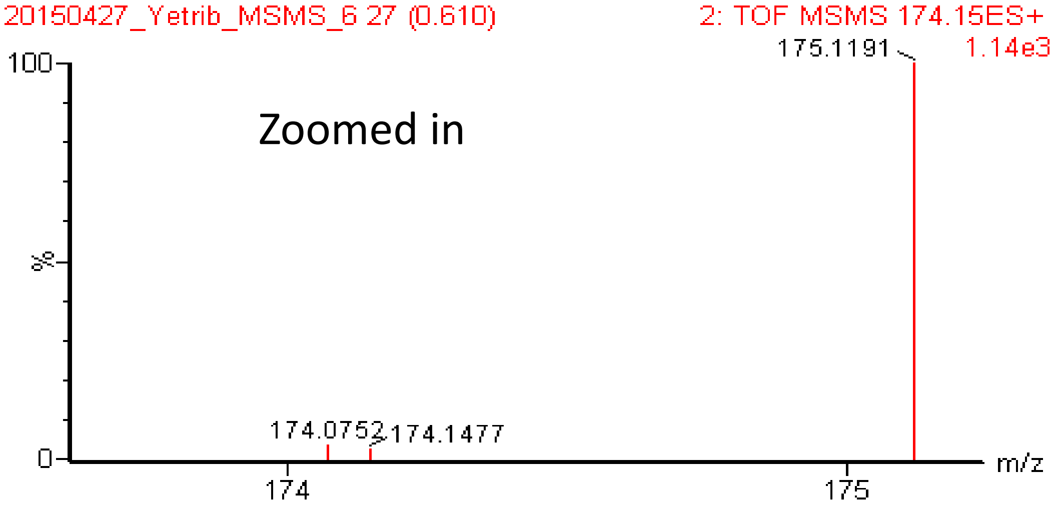
20150427_Yetrib_MSMS_6 27 (0.610)
2: TOF MSMS 174.15ES+
175.1191
1.14e3
Zoomed in
174.0752
174.1477
%
m/z

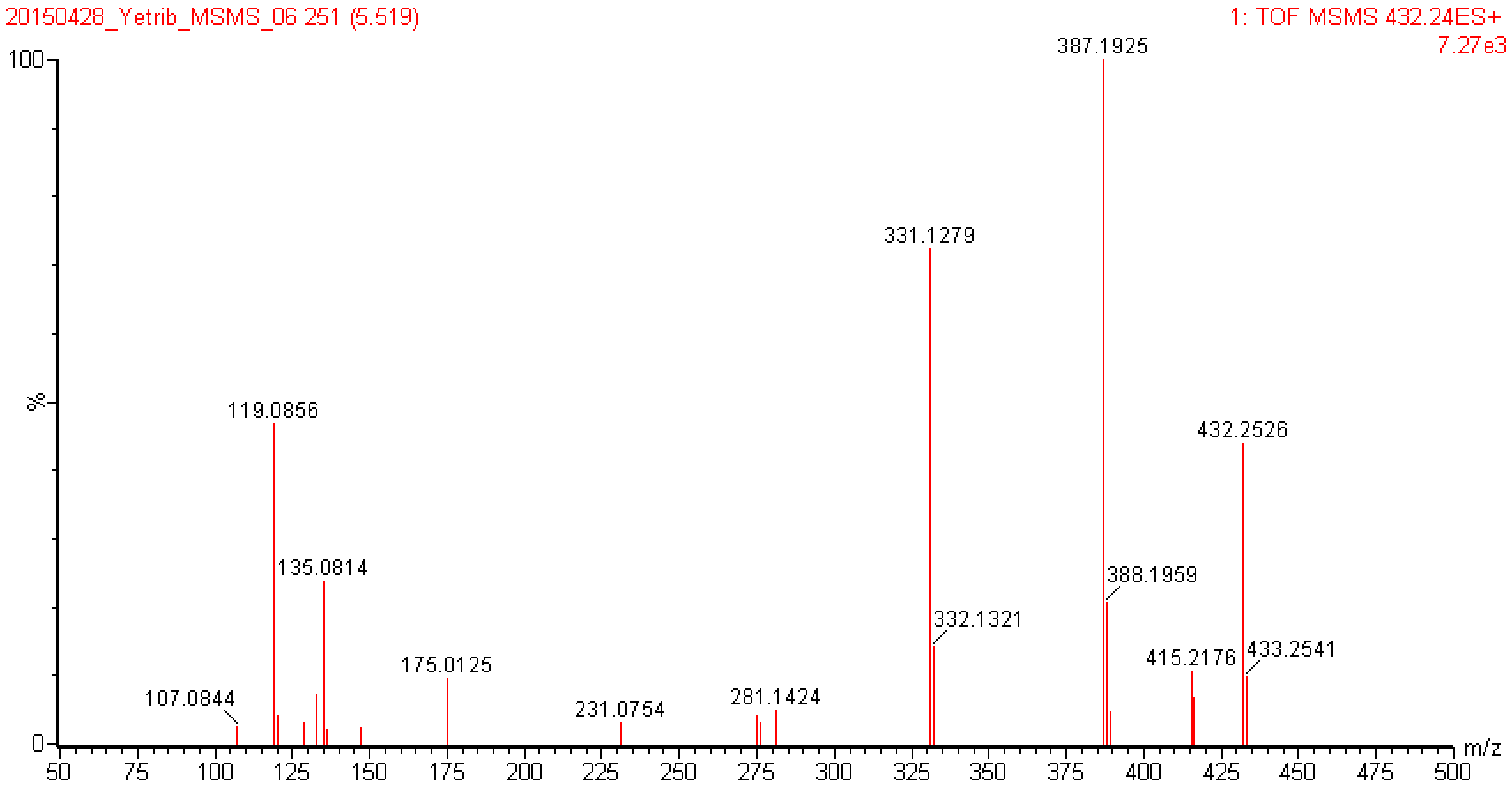
20150428_Yetrib_MSMS_06 251 (5.519)
1: TOF MSMS 432.24ES+
7.27e3
100
%
0
387.1925
331.1279
119.0856
432.2526
135.0814
388.1959
332.1321
415.2176
433.2541
175.0125
107.0844
281.1424
231.0754
50
75
100
125
150
175
200
225
250
275
300
325
350
375
400
425
450
475
500
m/z

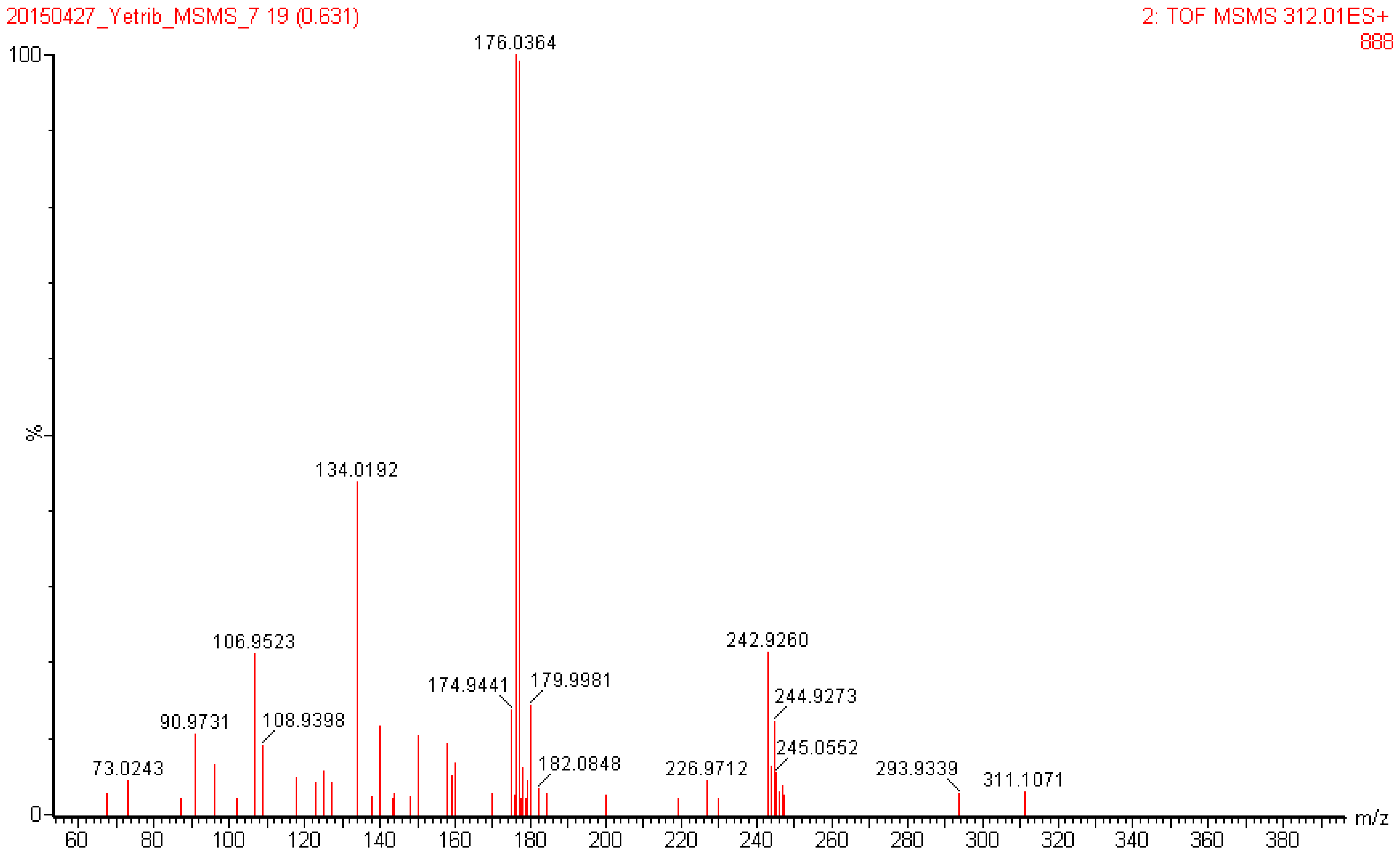
20150427_Yetrib_MSMS_7 19 (0.631)
2: TOF MSMS 312.01ES+
888
176.0364
134.0192
106.9523
242.9260
174.9441
179.9981
244.9273
90.9731
108.9398
245.0552
182.0848
73.0243
226.9712
293.9339
311.1071
100
%
0
60
80
100
120
140
160
180
200
220
240
260
280
300
320
340
360
380
m/z

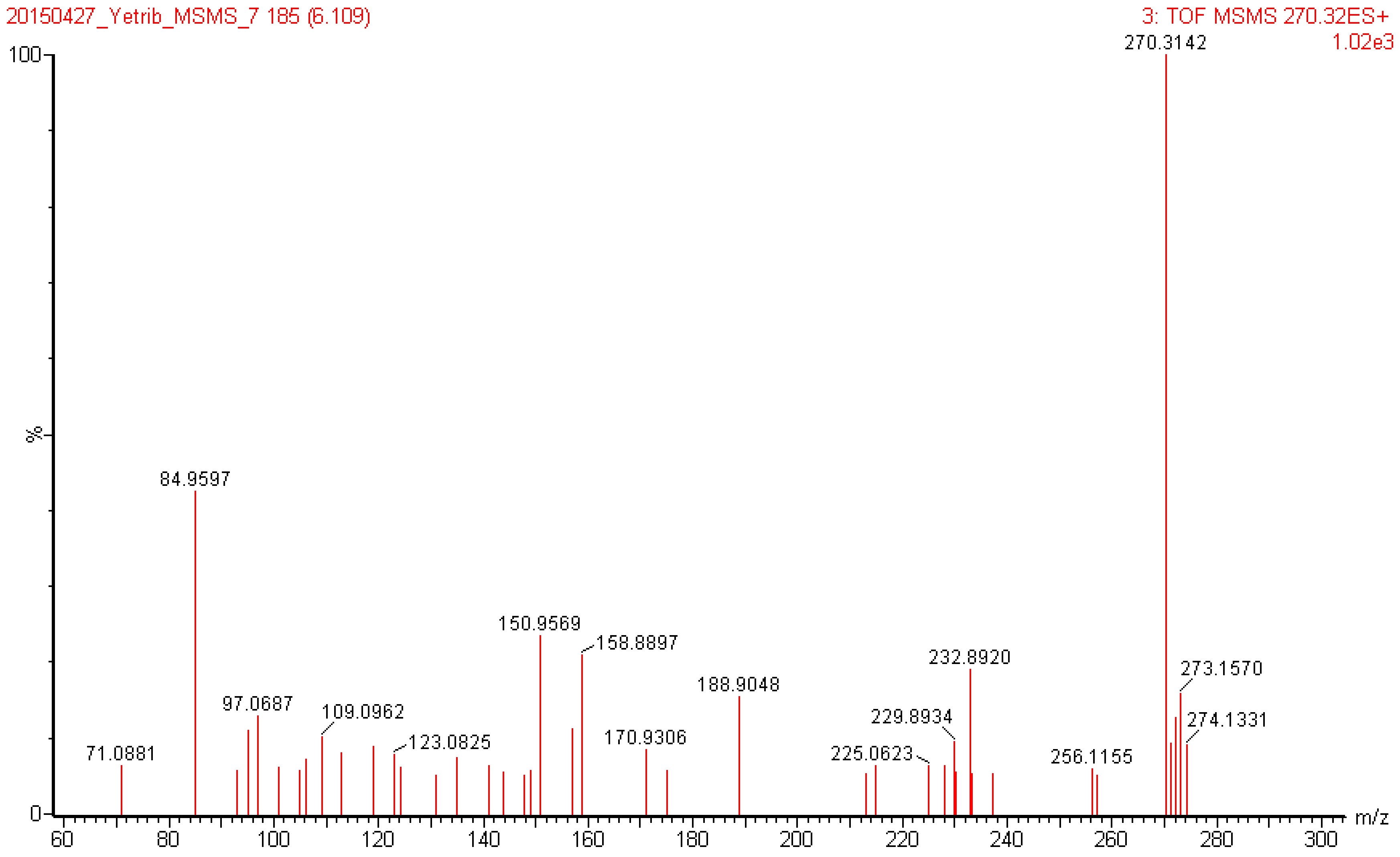
20150427_Yetrib_MSMS_7 185 (6.109)
3: TOF MSMS 270.32ES+
1.02e3
100
%
0
270.3142
84.9597
150.9569
158.8897
97.0687
109.0962
123.0825
71.0881
170.9306
188.9048
232.8920
229.8934
225.0623
256.1155
273.1570
274.1331
60
80
100
120
140
160
180
200
220
240
260
280
300
m/z

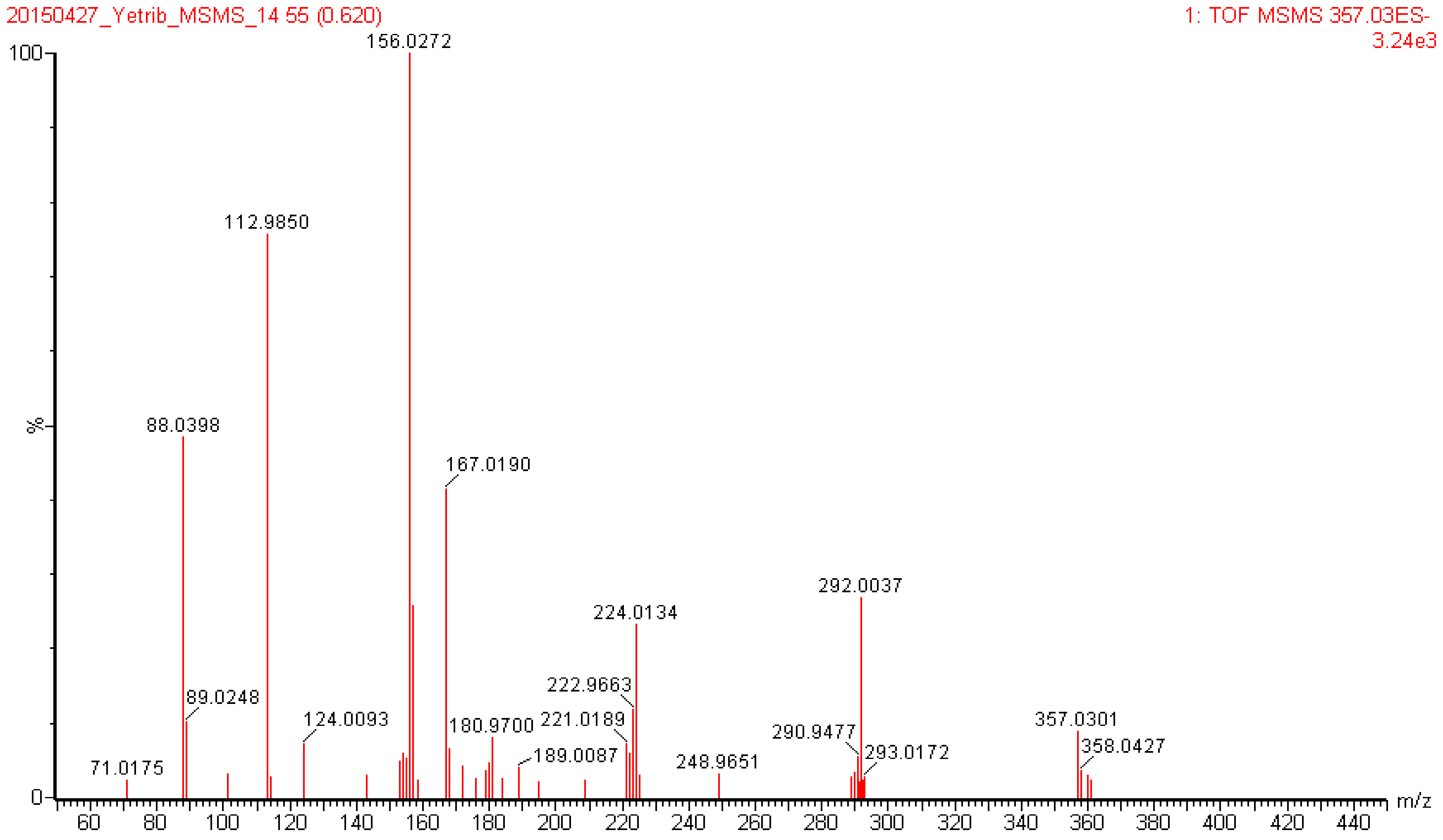

20150427_Yetrib_MSMS_14 55 (0.620)
1: TOF MSMS 357.03ES-
3.24e3
100
%
0
156.0272
112.9850
88.0398
167.0190
224.0134
292.0037
89.0248
124.0093
180.9700
221.0189
222.9663
189.0087
248.9651
290.9477
293.0172
357.0301
358.0427
71.0175
60
80
100
120
140
160
180
200
220
240
260
280
300
320
340
360
380
400
420
440
m/z

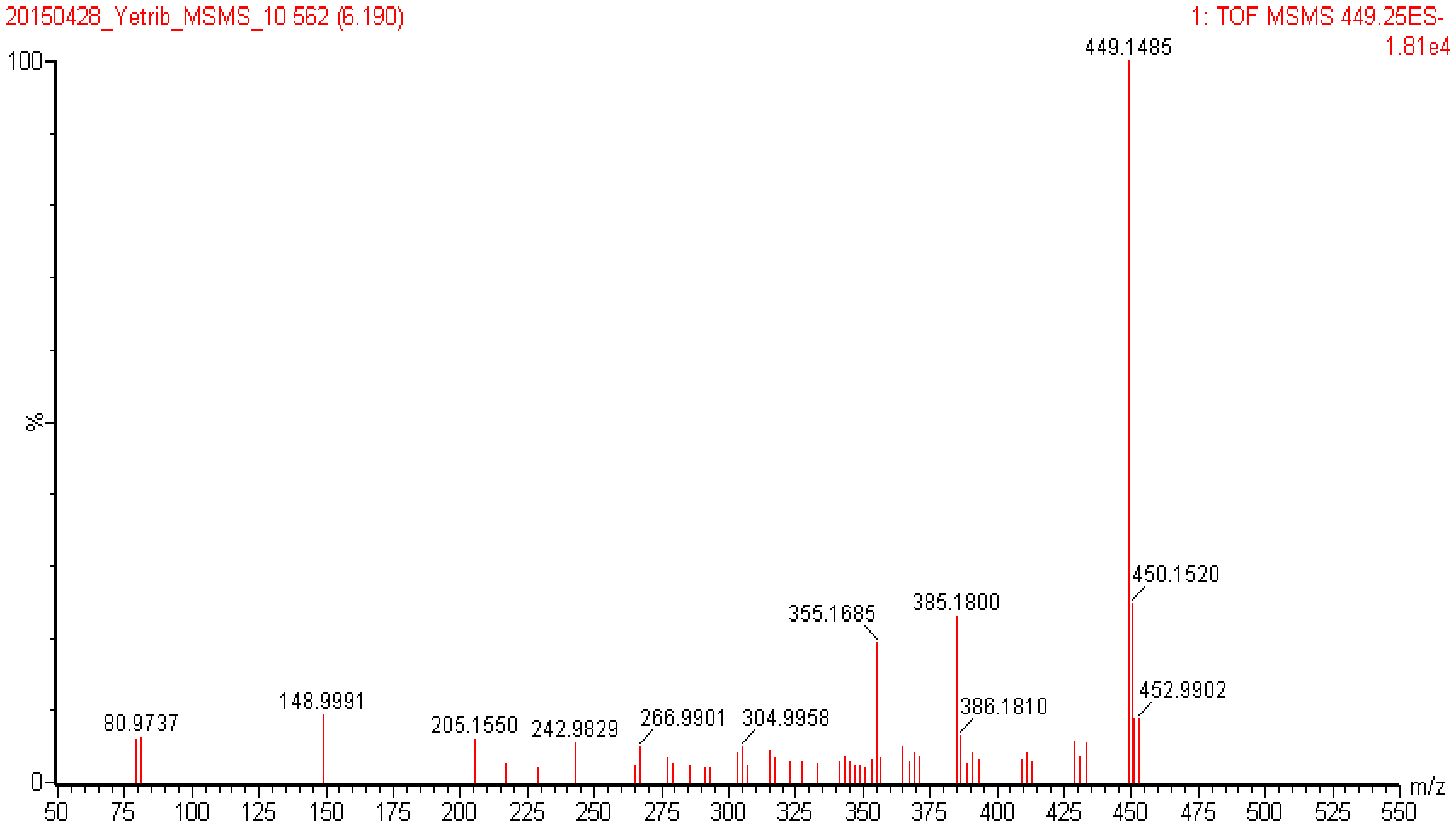
20150428_Yetrib_MSMS_10 562 (6.190)
1: TOF MSMS 449.25ES-
1.81e4
449.1485
450.1520
385.1800
355.1685
452.9902
386.1810
148.9991
80.9737
205.1550
242.9829
266.9901
304.9958
100
%
0
m/z
50
75
100
125
150
175
200
225
250
275
300
325
350
375
400
425
450
475
500
525
550
