# Supplementary figures and images for: Discovery of Metabolic Biomarkers for Duchenne Muscular Dystrophy within a Natural History Study
Source: PLoS One. 2016 Apr 15;11(4):e0153461. doi: 10.1371/journal.pone.0153461 (PMC4833348; doi:10.1371/journal.pone.0153461)

$\rho$  (overall) =  $-0.43$  ( $p = 0.0015$ )

$\rho$  (4–7 years) =  $-0.39$  ( $p = 0.15$ )

$\rho$  (>7–11 years) =  $-0.45$  ( $p = 0.26$ )

$\rho$  (>11–18 years) =  $-0.5$  ( $p = 0.042$ )

$\rho$  (>18–29 years) =  $-0.63$  ( $p = 0.037$ )

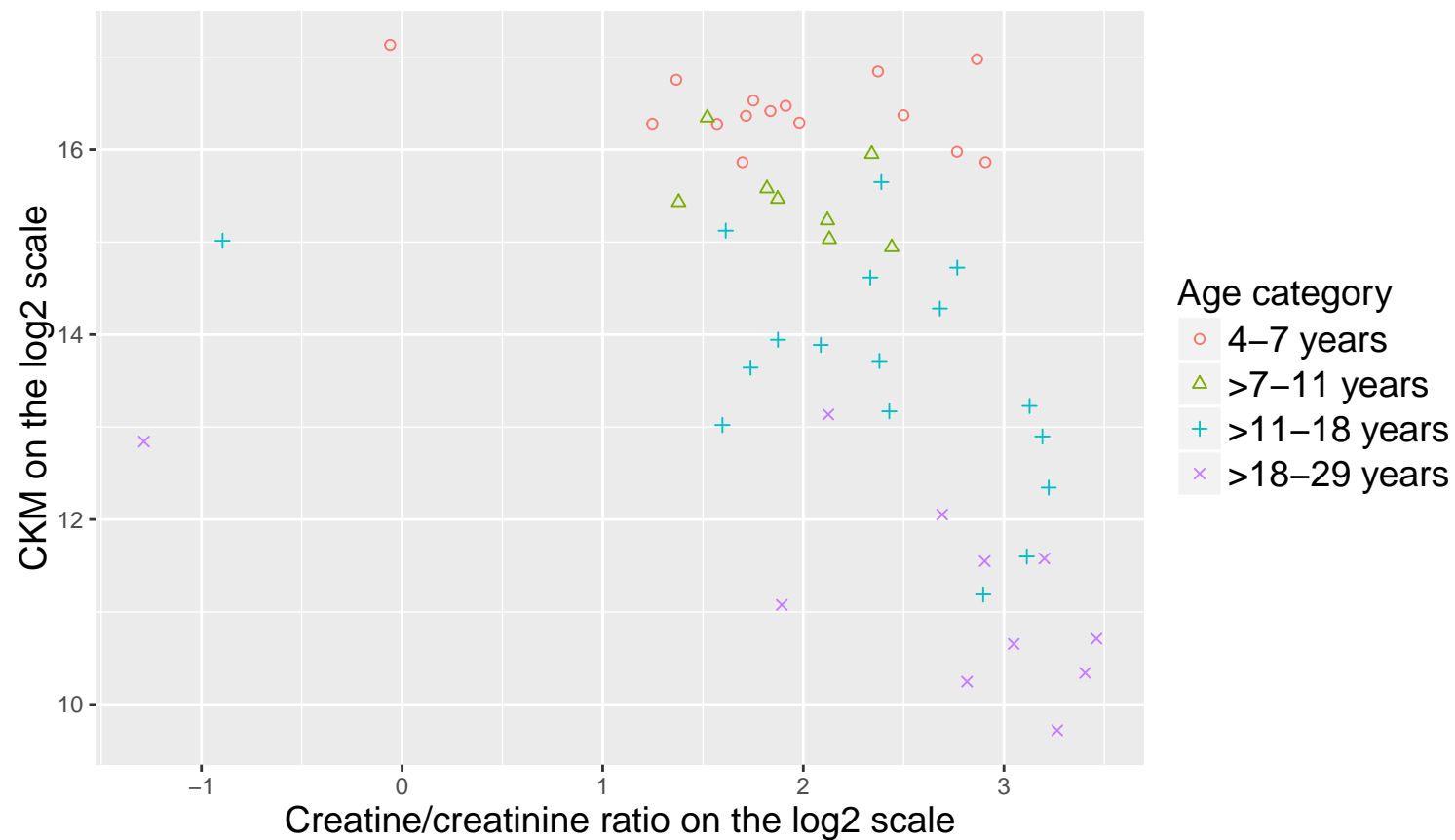

Supplement: S2 Fig — The intensity levels for creatine and creatinine have been internal standard normalized and quantile normalized. The points are color- and shape-coded by age category. ρ represents the correlation on the log scale. Both the overall correlation and the correlations within age categories are given. (PDF) [file pone.0153461.s002.pdf]
