# Supplementary material for: Discovery of Metabolic Biomarkers for Duchenne Muscular Dystrophy within a Natural History Study
Source: PLoS One. 2016 Apr 15;11(4):e0153461. doi: 10.1371/journal.pone.0153461 (PMC4833348; doi:10.1371/journal.pone.0153461)
Supplement: S3 Fig — A logistic model was fit with case/control status as outcome and age and peak intensity as explanatory variables. The AUC for each possible biomarker is given in the title; tpr = true positive rate, fpr = false positive rate. (PDF) [file pone.0153461.s003.pdf]

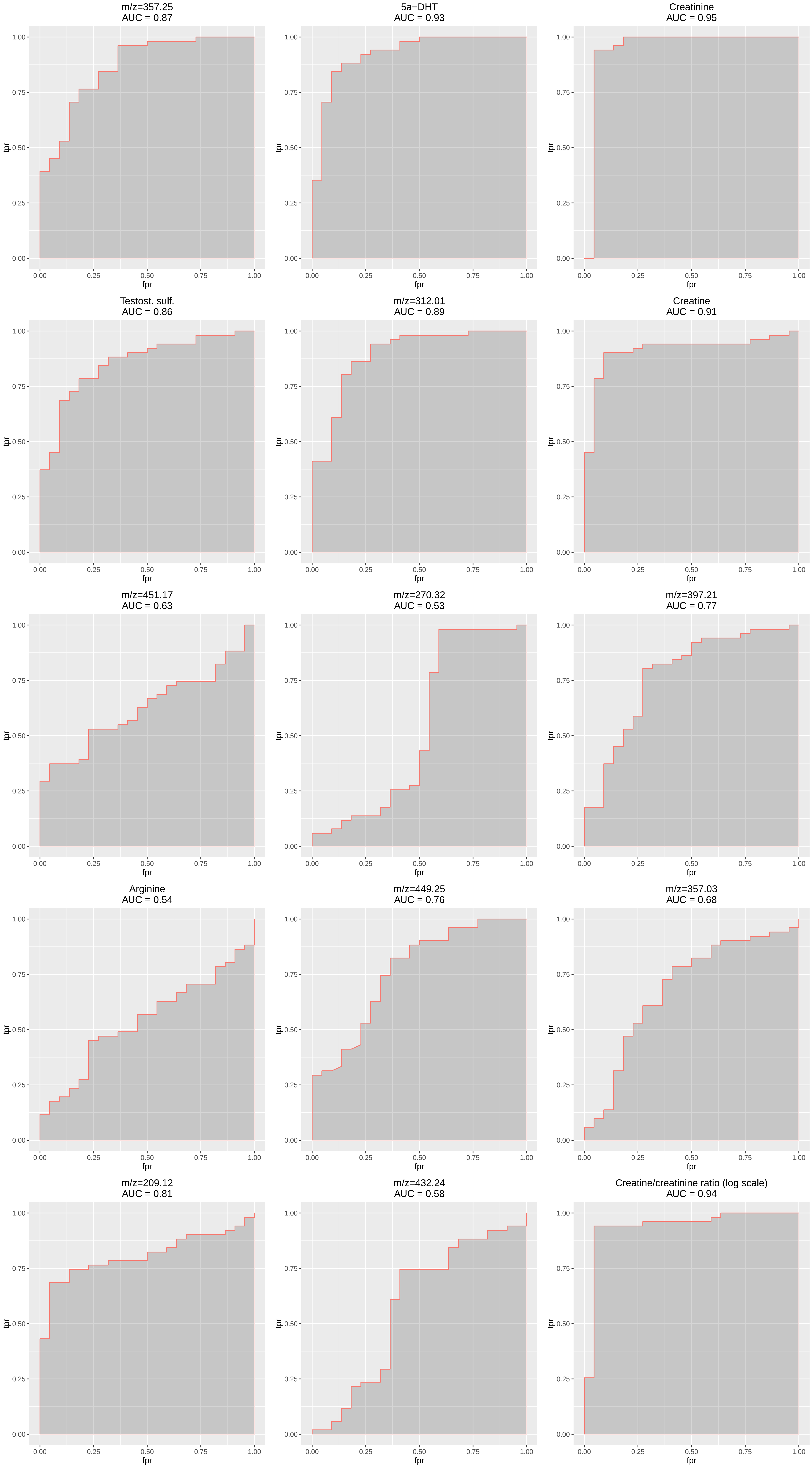
m/z=357.25
AUC = 0.87
5a−DHT
AUC = 0.93
Creatinine
AUC = 0.95
Testost. sulf.
AUC = 0.86
m/z=312.01
AUC = 0.89
Creatine
AUC = 0.91
m/z=451.17
AUC = 0.63
m/z=270.32
AUC = 0.53
m/z=397.21
AUC = 0.77
Arginine
AUC = 0.54
m/z=449.25
AUC = 0.76
m/z=357.03
AUC = 0.68
m/z=209.12
AUC = 0.81
m/z=432.24
AUC = 0.58
Creatine/creatinine ratio (log scale)
AUC = 0.94
tpr
fpr
0.00
0.25
0.50
0.75
1.00
